# Photophysical Processes of Porphyrin and Corrin Complexes with Nickel and Palladium

**DOI:** 10.3390/ijms27031577

**Published:** 2026-02-05

**Authors:** Maria Jaworska, Piotr Lodowski

**Affiliations:** Institute of Chemistry, University of Silesia in Katowice, Szkolna 9, 40-006 Katowice, Poland; piotr.lodowski@us.edu.pl

**Keywords:** porphyrin, corrin, nickel, palladium, DFT, TDDFT, NEVPT2

## Abstract

Nickel(II) and palladium(II) ions are capable of forming complexes with macrocyclic terapyrrole structures such as the porphyrin or corrin ring. Many different derivatives of these complexes are synthesized and studied because these compounds have potential numerous applications, including catalysis, various light-driven chemical reactions and processes related to intramolecular and intermolecular energy redistribution. Nickel porphyrins exhibit neither fluorescence nor phosphorescence when excited with light; however, palladium porphyrins, when excited to the singlet state, very quickly transform into the triplet state, and unlike nickel porphyrins, deactivation of the excited states occurs by phosphorescence. Palladium corrin has dual luminescent properties and exhibits both a weak fluorescence and strong phosphorescence. These photophysical differences are based on the complex energetic redistribution of singlet and triplet excited states interacting with each other in the intersystem crossing process. Based on the results of calculations at the DFT/TDDFT and CASSCF/NEVPT2 levels of theory, the structure of electronic excited states of model nickel(II) and palladium(II) complexes with corrin and porphyrin macro-rings was characterized and potential paths of photophysical processes leading to the occupancy of low-lying triplet states were described. In nickel complexes, very low-energy triplet states are the main cause of the rapid radiationless deactivation of excited states via triplet photophysical pathways.

## 1. Introduction

Porphyrin and corrin are the two tetapyrrole ligands of crucial importance in the metabolism of living beings [1]. Iron complexes with a porphyrin derivative form the so-called heme, which is a component of many enzymes that control important biological processes such as oxygen transport, oxidation reactions, electron transfer and many others [2]. Corrin macrocycle forms a complex with cobalt, producing vitamin B_12_. Vitamin B_12_ derivatives, called cobalamins, are components of enzymes that catalyze important biological processes [3]. They participate in DNA synthesis and the metabolism of fatty acids and amino acids. Cobalamin enzymes are required in animal metabolism, but plants do not utilize them. Humans have two B_12_ enzymes: methionine synthase and methylmalonyl-CoA mutase [4]. Replacing cobalt with nickel in cobalamin produces the so-called nibalamins [5] which may play a role as antivitamin B_12_ [6]. Antivitamins B_12_ are structurally modified derivatives of cobalamins that are inactive and can be used to investigate problems with the absorption or deficiency of this vitamin.

Many porphyrin complexes with metals other than iron have been synthesized and studied [7,8,9,10,11]. These compounds have numerous applications, including synthesis, catalysis, and medicine. For corrin, complexes with metals other than cobalt are not as numerous as for porphyrins. Examples include nickel, rhodium, palladium, copper, magnesium, and zinc [12,13,14,15,16,17,18,19].

Nickel porphyrin complexes have been studied extensively due to the interesting photophysical processes that occur within them [10,20,21,22,23,24,25,26,27,28,29,30,31,32,33,34,35,36,37,38,39]. Nickel porphyrins exhibit neither fluorescence nor phosphorescence when excited with light. From the singlet state, there is a rapid transition to the triplet state and a very rapid, radiationless deactivation to the ground state. The structure and properties of palladium corrin have been determined, but this compound has not been widely studied. Palladium porphyrins, when excited to the singlet state, very quickly transform into the triplet state, but unlike nickel porphyrins, deactivation of the triplet state occurs by phosphorescence [9,40,41,42]. In the triplet state, palladium porphyrins interact with oxygen to act as photoreceptors and therefore they are of great interest as oxygen concentration indicators with technological and medical applications and as compounds used in photodynamic therapy [43,44,45,46,47,48,49,50,51,52]. The metal complexes of porphyrin and corrin were also the subject of theoretical studies alongside experimental investigation [35,42,53,54,55,56,57].

In the electronic spectra of porphyrin derivatives, two main bands can be distinguished: the low-intensity Q band at approximately 500 nm and the high-intensity B band, or Soret band [58]. These two bands arise from transitions between four orbitals: the two highest occupied π and the two lowest unoccupied π orbitals. This is the so-called four-orbital Gouterman model [9,58]. The same pattern is found in metalloporphyrins, but in this case numerous transitions involving *d*-type orbitals also occur; however, they usually have zero or low intensity. The photophysics of metal porphyrin derivatives has been widely investigated [7,9,30,37,59]. The Q and B bands are often called S_1_ and S_2_, similar to free porphyrins, but in fact, transitions containing *d* orbitals lie below and in the middle, so this nomenclature is not particularly suitable to metalloporphyrins.

The subjects of this work are complexes of porphyrin and corrin with nickel and palladium. Our goal was to determine the spectroscopic and photophysical properties of nickel and palladium complexes with porphyrin and corrin by theoretical methods.

## 2. Results

### 2.1. Nickel Complexes

#### 2.1.1. Geometry and Electronic Structure of Nickel Porphyrin and Nickel Corrin

The structural formulas, atom coloring, atom numbers and nomenclature for the studied complexes are given in Section 4.5. As it can be seen in Table 1 the Ni-N bonds in nickel(II) corrin (NiCorr) are shorter than those in nickel(II) porphyrin (NiPor). The calculated bond lengths and angles agree well with the experimental ones.

**Figure 1:**
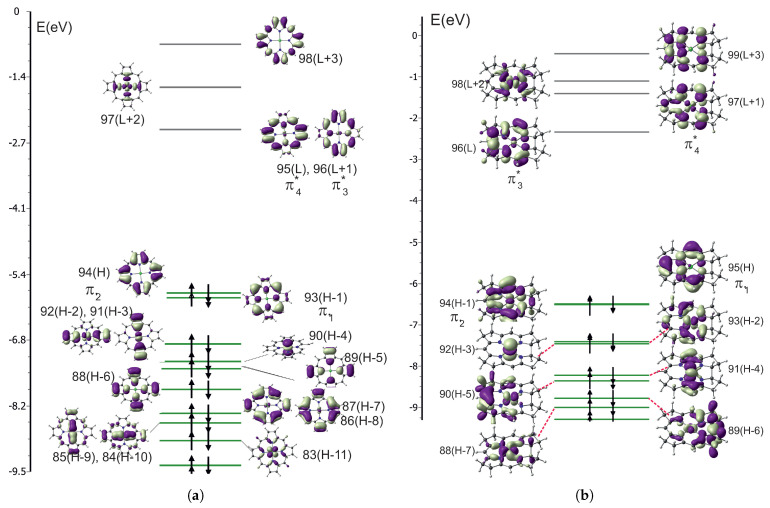
The orbital energy diagrams: (**a**) NiPor. (**b**) NiCorr.

In Figure 1, the MO diagrams of NiPor and NiCorr are depicted.

**Table 1:** Selected optimized geometry parameters of NiPor and NiCorr. Bond lengths in Å, angles in degrees. The experimental data are given for comparison. Atom numbering is shown in Section 4.5.

Parameter	NiCorr		NiPor	
	Calc.	Expt. ^a^	Calc.	Expt. ^b^
Ni-N_1_	1.848	1.851	1.958	1.930
Ni-N_2_	1.895	1.913	1.958	1.930
Ni-N_3_	1.895	1.884	1.958	1.930
Ni-N_4_	1.848	1.847	1.958	1.930
N_1_-Ni-N_2_	91.4	91.2	90.0	90.0
N_2_-Ni-N_3_	95.1	95.5	90.0	90.0
N_3_-Ni-N_4_	91.4	91.4	90.0	90.0
N_4_-Ni-N_1_	82.6	83.0	90.0	90.0
N_1_-Ni-N_3_	171.6	170.4	180.0	179.7
N_2_-Ni-N_4_	171.6	169.5	180.0	179.7
N_1_-N_2_-N_3_-N_4_	−7.2	−10.2	0.0	0.5
N_1_-N_2_-N_3_-Ni	−3.6	−4.7	0.0	0.2

^a^ Ref. [18], ^b^ Ref. [60]

#### 2.1.2. Electronic Spectra of NiPor and NiCorr

*A. NiPor*

The TDDFT calculated electronic transitions are gathered in Table 2 together with the experimental values. The four lowest energy singlet electronic transitions are of the d→d type with some π orbital contribution. These are symmetry-forbidden transition and the corresponding oscillator strengths are zero. The two transitions at 474 nm originate from excitation between four Gouterman orbitals and they correspond to the Q band. Comparison with the experimental data in Table 2 shows that this band for nickel porphyrins lies in the range 520–540 nm and reveals that the calculations give too high transition energies in the Q band. The two degenerate transitions at 325 nm again originate from excitations between the four Goutermann orbitals and correspond to the B (Soret) band. Calculations again give these transitions at shorter wavelengths than the experimental one.

From the perspective of the photophysical properties of nickel porphyrin derivatives, triplet states are very important. These compounds typically exhibit neither fluorescence nor phosphorescence. It is known that upon excitation to the singlet state in the Q or Soret band, a transition to the triplet state occurs, ultimately leading to fast radiationless deactivation through a d→d triplet state. Table 2 shows that the first three triplet transitions occur at very low energies, 0.3–0.6 eV. These are d→d transitions. The next transition occurs at 1.55 eV, also of the d→d type.

Subsequent transitions are $\pi \to \pi^{*}$, and from 3.0 eV onwards, mixed π→d and $d\to \pi^{*}$ transitions occur. The presence of low-energy triplet states is important for the radiationless deactivation of nickel porphyrin. Calculations show that these are d→d states. As will be shown later (Section 2.5), similar energies of triplet states are obtained by the multi-configuration NEVPT2 method.

*B. NiCorr*

The electronic spectra of nickel corrin were measured, as were the spectra of the derivatives of nickel B_12_ in which the cobalt was replaced by nickel, called nibalamin [5]. On the other hand, the photophysical properties of nickel corrins have not been studied experimentally.

The calculated electronic transitions of NiCorr are summarized in Table 3, along with the available experimental data. The four lowest singlet transitions have d→d character and zero or near-zero oscillator strengths. The non-zero oscillator strengths of some of the corrin derivatives are due to the ring non-planarity. For this reason, *d* orbitals generally mix with π orbitals in metallocorrins. Two transitions with high oscillator strengths are calculated at 364 and 345 nm. They have $\pi \to \pi^{*}$ character with a small admixture of *d* character. The experimental bands in the spectra of nickel corrin derivatives differ slightly in wavelength. They can be gathered into several groups: 450–460 nm, 416–435 nm, 319–340 nm, 294–317 nm, 266–277 nm, and 244–245 nm. These can be assigned to calculated transitions with significant oscillator strengths, at 364 nm, 345 nm, 284 nm, 271 nm, 256 nm, and 140 nm, respectively. These are essentially π→π transitions, with some *d* character. The electronic transition at 364 nm with a large oscillator strength is a pure $\pi \to \pi^{*}$ form and can be attributed to the experimental bands at 450–460 nm.

It can be seen that the calculated wavelengths of the electronic transitions are shorter than the experimental ones, similar to NiPor.

The lowest four triplet transitions have energies ranging from 1.18 eV to 2.30 eV, hence they have much higher energy than those in NiPor where the lowest triplet transition has an energy of 0.35 eV.

#### 2.1.3. Minimum Energy Crossing Point of T_1_ and S_0_

**Figure 2:**
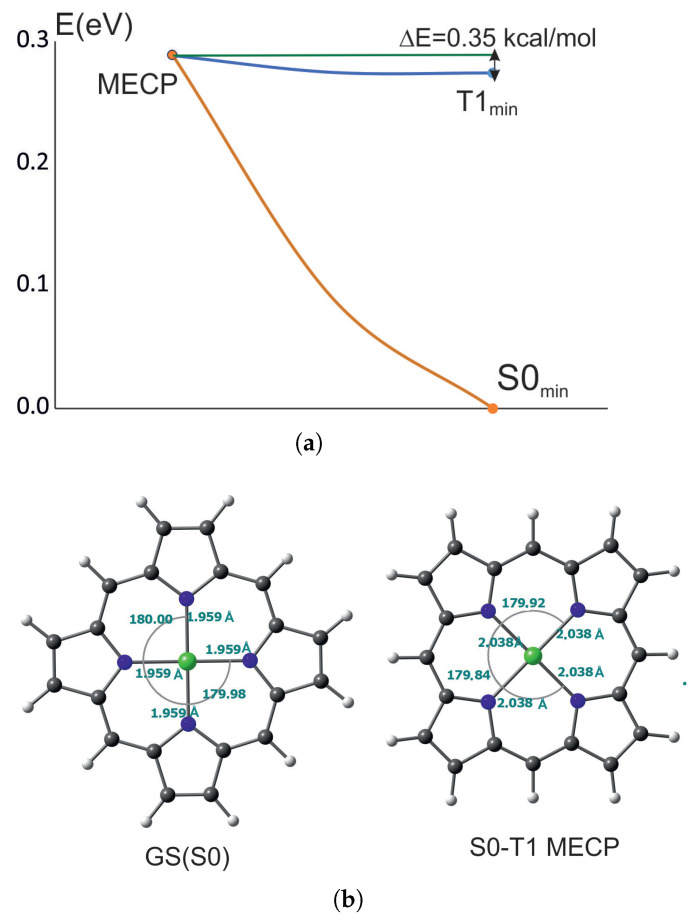
Minimum energy crossing point for NiPor: (**a**) Calculated energy barrier. (**b**) Comparison of the ground state (GS) and MECP geometries. q denotes general coordinate comprising angle and bond length change.

**Figure 3:**
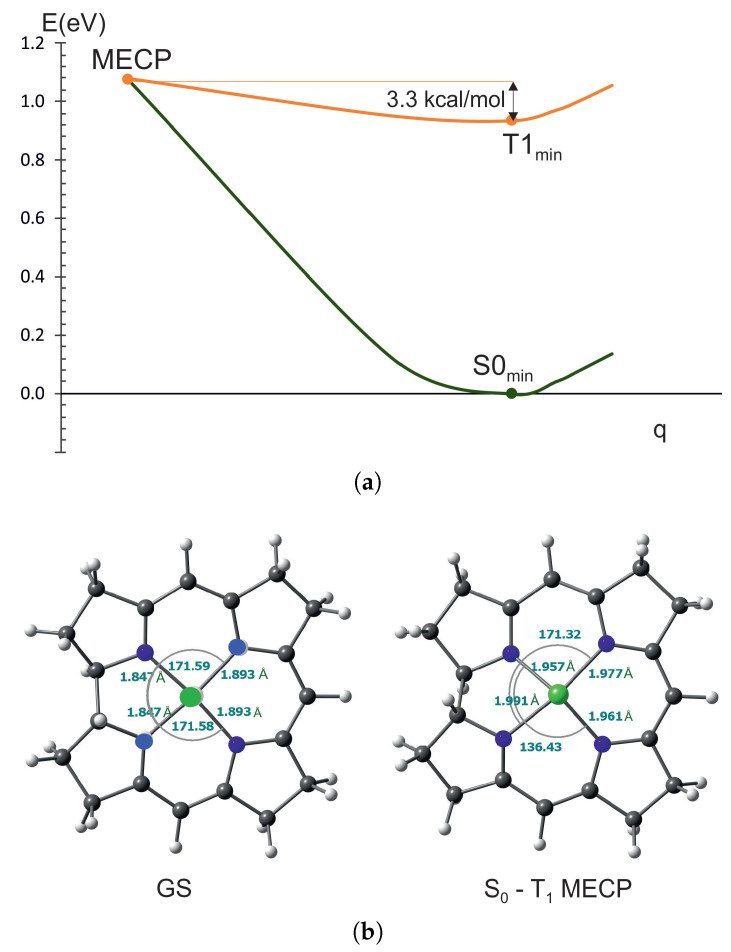
MECP between T_1_ and S_0_ for NiCorr: (**a**) The energy barrier between crossing point and triplet state minmum. (**b**) The comparison between S0 and MECP geometry for NiCorr.

The minimum energy crossing point (MECP) is the lowest energy point between two electronic states with different multiplicity. The MECPs between the lowest energy triplet state (T_1_) and the ground state were determined for NiPor and NiCorr. They are presented in Figure 2 and Figure 3. As can be seen, reaching the MECP from the triplet state minimum for NiPor requires only 0.35 kcal/mol, which is a very small barrier and the triplet transition to the ground state process should be very fast, which is in agreement with the experiment where it occurs within 200 ps [29,31,33].

In nickel porphyrin, the dihedral angles of the nickel ion practically do not change at the MECP point, but the nickel–nitrogen bonds are lengthened by about 0.07 Å. Therefore, the transition from the lowest triplet state to the ground state takes place by stretching the Ni-N bonds.

The situation is different in nickel corrin. The energy required to reach the MECP from the minimum of T_1_ state is 3.3 kcal/mol. The Ni-N bonds lengthen at the MECP by 0.7 to 0.12 Å, but one dihedral angle also changes, from 171 to 136 degrees. So, in this case, the return to the ground state is essentially through the ring bending apart from nickel–nitrogen bond elongation. In the case of nickel complexes, the lowest triplet states are d→d and their energy is very low, especially in nickel porphyrin. Therefore, the transition to the ground state involves a very small energy barrier. Experimentally, it is believed that the transition from the triplet state to the ground state in nickel porphyrins involves a state of d→d character [31,61], which is in agreement with the calculations.

### 2.2. Palladium Complexes

#### 2.2.1. Geometry and Electronic Structure of Palladium Porphyrin and Palladium Corrin

Optimized geomety parameters of PdPor (Palladium(II) Porphyrin) and PdCorr (Palladium(II) Corrin) are gathered in Table 4. The calculated palladium–nitrogen bond lengths in PdCorr are shorter than the experimental ones. On the other hand the respective bond lengths in PdPor agree very with the experimental ones. As can be expected, the palladium–nitrogen bonds are longer than those in the nickel complexes.

**Figure 4:**
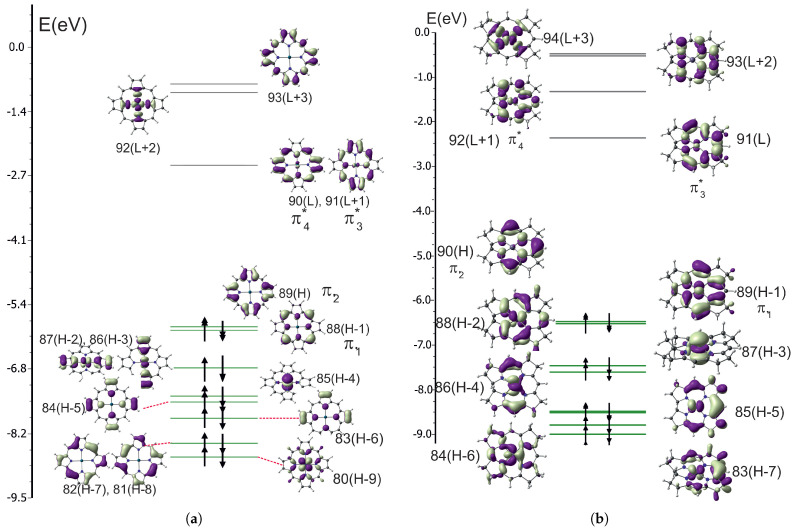
Molecular orbital diagrams: (**a**) PdPor. (**b**) PdCorr.

The MO diagrams for PdPor and PdCorr are shown in Figure 4.

For PdPor, similarly as for NiPor, the two highest occupied and two lowest unoccupied orbitals are π-type Gouterman orbitals. For PdCorr those orbitals are also π-type, but some admixture of *d* orbitals is visible.

#### 2.2.2. Electronic Spectra for Palladium Porphyrin and Palladium Corrin

*A. PdPor*

The TDDFT calculated electronic transitions for PdPor are collected in Table 5, along with the experimental data. The lowest two transitions at 468 nm are degenerate and correspond to the Q band. These are $\pi \to \pi^{*}$ type transitions involving the two highest occupied orbitals and the two lowest unoccupied ones, the four Goutermann orbitals. The experimental bands are given for various derivatives and range between 512 and 524 nm. The calculated transitions are at shorter wavelengths than the experimental ones, as in nickel complexes.

The Soret band is calculated at 326 nm (experimentally between 394 and 417 nm). It also derives from excitations between the Goutermann orbitals. Six transitions, all of $d\to \pi^{*}$ type, appear between the Q and Soret bands in the calculations. Above the Soret band, $d\to \pi^{*}$ and d→d transitions appear, and at higher energy, $\pi \to \pi^{*}$ transitions again, involving orbitals outside the four orbitals that make up the Q and Soret bands. We can see here that the d→d transitions occur in palladium porphyrin at much higher energies than in nickel porphyrin. Their oscillator strengths are equal to zero, because of the high symmetry of the porphyrin ring. The triplet states start at 2.09 eV, and the four lowest energy ones are of $\pi \to \pi^{*}$ character. The electronic triplet states involving *d* orbitals start at 2.94 eV. Here we can see the large difference from nickel porphyrin.

*B. PdCorr*

Palladium corrin derivatives have been synthesized and their electronic spectra measured. However, data on the photophysics of these systems are relatively scarce, and they have not attracted as much attention as palladium porphyrins.

Due to the non-planar ring structure (bent N-Pd-N bonds, Table 4), the π and *d* orbitals mix, and consequently, bands such as Q and Soret cannot be distinguished. Two bands at long wavelengths of 460 nm and 430–440 nm were experimentally identified. The electronic transitions calculated using the TDDFT method are gathered in Table 6.

The two lowest transitions are of the $\pi \to \pi^{*}$ type, the second one containing some d character. These can be assigned to experimental transitions at 460 and 430–440 nm. Subsequent transitions are of the $d\to \pi^{*}$ and d→d type, up to the transition at 273 nm, which has $\pi \to \pi^{*}$ character. In this respect, there is a similarity to palladium porphyrin—the $\pi \to \pi^{*}$ transitions are separated by $d\to \pi^{*}$ and π→d transitions. At higher energies, mixed π→d, $d\to \pi^{*}$, and d→d transitions occur.

The triplet states show a similar pattern, the lowest two are $\pi \to \pi^{*}$ states, then come the mixed $d\to \pi^{*}$, π→d and d→d states; at 270 nm a $\pi \to \pi^{*}$ state appears again.

#### 2.2.3. MECP for Palladium Complexes

**Figure 5:**
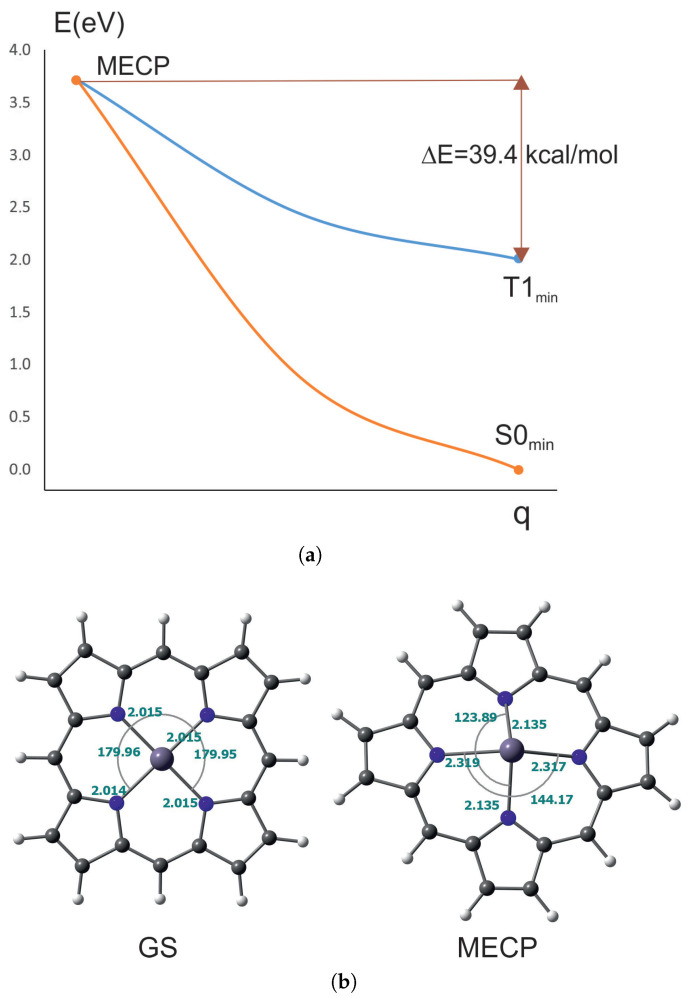
Palladium(II) porphyrin: (**a**) Energy barrier of MECP in PdPor. (**b**) Comparison of the geometry of MECP and the ground state.

In Figure 5, the T_1_/S_0_ crossing is shown for PdPor. The geometry of palladium porphyrin in the MECP point shows a large deformation within the palladium coordination sphere, with dihedral angles of 123 and 144 degrees. The Pd-N bonds elongate in pairs to 2.1 and 2.3 Å. The molecule forms an asymmetric dome. The energy required to transition from the T_1_ state minimum to the MECP point is 39.4 kcal/mol. Such a high barrier is unfeasible to reach by the molecule, and deactivation to the ground state occurs through phosphorescence. One can note that the T_1_ state is a $\pi \to \pi^{*}$ state belonging to the Q band, which distinguishes palladium porphyrin from nickel porphyrin and accounts for the high triplet–singlet crossing barrier.

**Figure 6:**
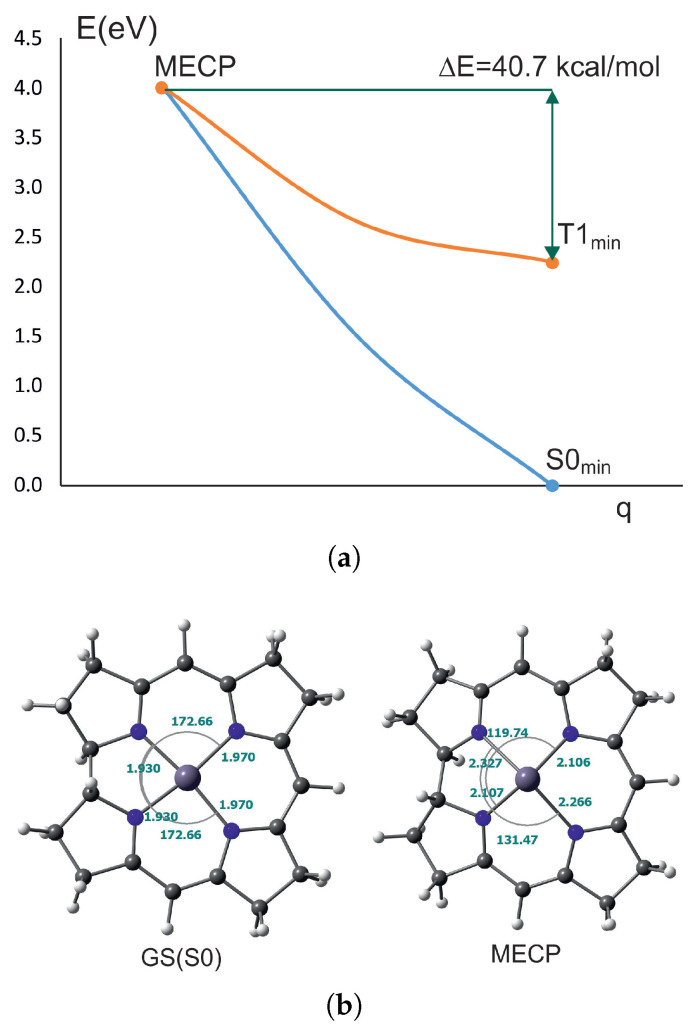
Minimum energy crossing point for PdCorr: (**a**) Energy barrier between MECP and triplet state minimum (**b**) Comparison of the ground state and MECP geometry.

The MECP for T_1_/S_0_ crossing for PdCorr is shown in Figure 6. In palladium corrin, the situation regarding the MECP geometry is very similar to palladium porphyrin. The valence angles within the coordination sphere change substantially compared to the ground state, to 119.7 and 131.4 degrees, and the bonds lengthen to 2.1–2.3 Å. The molecule takes the form of an irregular dome with the palladium atom protruding above its plane (or rather, in this case, above the plane of the four nitrogen atoms). The energy barrier is similar to PdPor and amounts to 40.7 kcal/mol.

### 2.3. Emission of Nickel and Palladium Complexes

The calculated and experimental fluorescence and phosphorescence wavelengths for nickel and palladium complexes with porphyrin and corrin are collected in Table 7.

Palladium porphyrins exhibit phosphorescence at wavelengths above 600 nm, the exact value depending on the porphyrin form and the nature of the fluorescence. Very weak fluorescence above 500 nm is also observed, the appearance of which is influenced by the type of solvent. The calculated phosphorescence wavelength is 703 nm, while the fluorescence wavelength is calculated to be 475 nm. The phosphorescence wavelength is slightly longer than the experimental wavelength, while the fluorescence wavelength is shorter. When converted to energy units, this gives differences of approximately 0.35 eV for fluorescence and 0.1 eV for phosphorescence.

In the case of nickel porphyrin and nickel corrin, virtually no emission is observed. Excitation to a singlet and a rapid transition to the triplet state are followed by a very fast transition to the ground state. This situation is favored by the low-lying triplet states in these complexes, as shown by the results of TDDFT and NEVPT2 calculations (Section 2.5). However, in a study for nickel porphyrin derivatives NiTMP and NiSWTP, the molecule was excited to the Q band, which led to ultrafast stimulated emission at a wavelength of 620–670 nm [33]. No fluorescence was observed upon excitation to the Soret band, leading to the conclusion that the ISC to the triplet state from this band is faster than the IC to the lower singlet states. However, from the triplet states, there is rapid deactivation to the ground state. The calculated fluorescence wavelength is 708 nm, which agrees well with the experiment.

### 2.4. Spin–Orbit Interaction in Nickel and Palladium Porphyrin and Corrin

For the transition between singlet and triplet states to occur, the integral of the spin–orbit coupling operator, $H_{ST}^{SO}$, must be large. These states also have to be close in energy to enable their intersection (Intersystem Crossing, ISC). The SOC coefficients (SOCCs) were determined for the singlet and triplet states of NiPor, NiCorr, PdPor, and PdCorr. In Table 8, the calculated values with the TDDFT method are gathered. Only values larger than 100 cm^−1^ are shown. They are also summarized in Tables S2–S6 in Supplementary Materials.

### 2.5. NEVPT2 Calculations

The singlet and triplet transition energies for NiPor calculated using the NEVPT2 method are summarized in Table 9. The natural CASSCF orbitals are presented in Figure S2 (Supplementary Materials). The lowest few singlet transitions are of the $d\to \pi^{*}$, d→d, and π→d type. They have zero oscillator strengths, which results from the symmetry of the porphyrin ligand. Two degenerate transitions at 517 nm involving four π orbitals can be assigned to the Q band. The wavelengths agree well with the experimental data (see Table 2). The oscillator strengths of these transitions are low, which is also consistent with the experiment. Next, the calculations indicate three transitions: one d→d and two $d\to \pi^{*}$.

These are followed, at 387 nm, by two $\pi \to \pi^{*}$ transitions with high oscillator strengths, corresponding to the Soret band. The four lowest triplet transitions are of the d→d type, and three of them have very low energies, 0.2–0.3 eV, similar to those determined by the TDDFT method (Table 2).

The electronic transitions calculated with the NEVPT2 method for NiCorr are collected in Table 10. The CASSCF natural orbitals are presented in Figure S3 (Supplementary Materials). The calculated transitions with wavelengths from 570 to 490 nm are of the d→π and d→d types. At 462 nm, a transition with a high oscillator strength occurs and has a $\pi \to \pi^{*}$ character. This can be attributed to the experimental transition at around 460 nm. The next two transitions with a high oscillator strength occur at 342 and 305 nm, which also compare well with the experimental transitions. These are $\pi \to \pi^{*}$ transitions with an admixture of $d\to \pi^{*}$ character. As in the TDDFT results, there is a large mixing of transitions containing *d* orbitals with $\pi \to \pi^{*}$ transitions, which is a consequence of the non-planar structure of the corrin ring.

When considering triplet states, the three lowest ones have very low energy, 0.4–0.6 eV, and are of d→d type. Such low-energy triplets also occur in NiPor. At shorter wavelengths, mainly $d\to \pi^{*}$ transitions occur, and at 484 and 406 nm, $\pi \to \pi^{*}$ transitions are found.

The electronic transitions calculated with the NEVPT2 method for PdPor are collected in Table 11. The CASSCF natural orbitals are shown in Figure S4 (Supplementary Materials). In Table S8, the lowest singlet and triplet electronic transitions for PdPor complex calculated with the CASSCF(14,13)/NEVPT2/def2-TZVP with the occupation numbers of all active orbitals are gathered. For PdPor, in the spectrum determined by the NEVPT2 method, the Q band occurs at 507 nm and the Soret band at 380 nm. Several $d\to \pi^{*}$ transitions occur between these bands. Above the Soret band, transitions involving *d* orbitals appear, namely, $d\to \pi^{*}$, d→d, and π→d. Triplet states begin at 579 nm. The lowest are $\pi \to \pi^{*}$ transitions; at higher energies, states involving *d* orbitals are found.

The electronic transitions calculated for PdCorr by the NEVPT2 method are gathered in Table 12. The CASSCF natural orbitals are depicted in Figure S5 (Supplementary Materials). The NEVPT2 spectrum for PdCorr shows two intense transitions at 382 and 368 nm, which can be assigned to the experimental bands at 460 and 420–430 nm. It can be seen that the calculated wavelength is shorter than the experimental one in this case. These transitions are of $d\to \pi^{*}$, $\pi \to \pi^{*}$, and d→d character. In addition, there are two intense transitions at 299 nm and 274 nm, which correspond to the position of the intense bands in NiCorr (see Table 3). Their character is similar to the earlier transitions. As in NiCorr, the $\pi \to \pi^{*}$ transitions are mixed with transitions involving *d* orbitals.

## 3. Discussion

### 3.1. Photophysics of Nickel and Palladium Porphyrin and Corrin

#### 3.1.1. NiPor and PdPor

**Figure 7:**
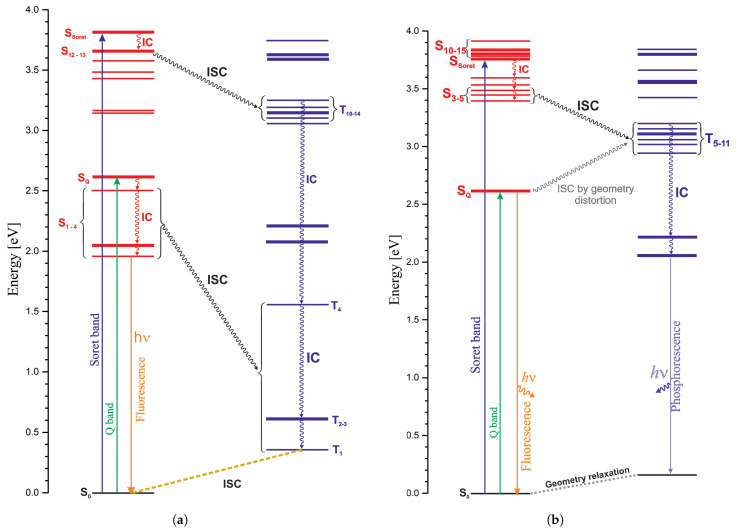
Schematic representation of porphyrin complexes’ photophysics: (**a**) NiPor. (**b**) PdPor.

The proposed photophysical processes in NiPor and PdPor are described in Figure 7. They were made based on the calculated electronic spectra and spin–orbit coupling coefficients (SOCCs). Figure 7a depicts a diagram for NiPor. The groups of singlet and triplet states for which the SOCCs are large are marked with a wavy line.

It has been found that upon excitation to the singlet state, a rapid transition to the triplet state occurs in NiPor [26,38,65]. This may be the close-lying $T_{n}$ triplet, from which the system comes to T_1_ through a series of internal conversions. This vibrationally excited state is mediated by vibrational relaxation, and from this state, a rapid transition to the singlet ground state occurs. This process takes approximately 200 ps. In solutions containing molecules with coordinating properties, such as pyridine, they bind to nickel porphyrin in the excited triplet state, forming five- or six-coordinate complexes [27,30]. These ligands dissociate upon transition to the nickel porphyrin ground state. It is usually considered that the transition to the triplet state occurs from the Q band and if the excitation takes place at the Soret band, there is at first an internal conversion (IC) to the lower Q band, then through intersystem crossing (ISC) to the triplet. There is agreement that the transition to the ground state occurs from a state of d→d nature. Nickel porphyrins have no emissive properties and do not exhibit fluorescence or phosphorescence; however, stimulated fluorescence was obtained for NiTPP by exciting it to the Q band. This gave a fluorescence signal of 1 ps. Emission occurs at 620–670 nm. Fluorescence was not observed after excitation to the B (Soret) band, which leads to the conclusion that the transition from the Soret band to the triplet state (ISC) is faster than the internal conversion to the Q state.

Excitation of NiPor to the Q state (calculated at 2.61 eV, Table 2) leads to a rapid transition to the d→d and π→d states lower in energy (S_1−3_). The S_2_ and S_3_ states (2.04 eV) have large SOC coefficients (see Table 8) with the T_4_ state of energy equal to 1.55 eV. Considering the relatively small energy difference between S_2,3_ and T_4_, a rapid singlet–triplet transition (ISC) may occur, followed by a rapid internal conversion to the T_1_ state, and then an ISC to the ground state (S_0_). Note that the S_1_ state has a high SOCC only with the T_1_ state, but the energy difference between them is large (1.6 eV) enough that such a transition is unlikely. Hence, rapid fluorescence may occur if the system reaches the S_1_ state instead of the conversion to triplet state. From T_1_ to S_0_, the energy barrier is very small, 0.35 kcal/mol (Figure 2). The state T_1_ has a d→d character, hence confirming the experimental findings that deactivation of excited states in nickel porphyrin occurs through triplet states, with the last step estimated at 200 ps.

If we consider excitation to the B (Soret) state, the situation can be depicted as follows: $S_{0}\overset{IC}{\to }S_{12,13}\;\left(3.65\;eV\right)\overset{ISC}{\to }T_{13,14}\;\left(3.2\;eV\right)\overset{IC}{\to }T_{1}\overset{ISC}{\to }S_{0}$. The S_12,13_ and T_13,14_ states have an energy difference of approximately 0.4 eV and high SOC coefficients. A transition (ISC) between them is possible in this case. From the T_12,13_ states, a rapid transition (IC) occurs to the T_1_ state and then ISC to the S_0_ state. Experimentally, it has been shown that although rapid fluorescence can be detected upon excitation to the Q state, it is absent upon excitation to the B state, which leads to the conclusion that the ISC process from the B state to a triplet is faster than the IC process to the lower lying singlets. There is a large energy gap between the B state and its neighboring states and the Q state, which may explain such a situation.

Palladium porphyrins are intensively studied for their applications in medicine and technology. In both cases, their phosphorescence ability is utilized, as palladium porphyrins, upon excitation to a singlet state, transfer to a triplet state by intersystem crossing process (ISC) and from triplet to the ground state through phosphorescence. When inspecting the PdPor diagram in Figure 7b, one can see a significant difference from NiPor.

The Q state is the lowest-energy state, and the SOCC values for Q and the T_1−4_ states are zero. This is understandable, as these are $\pi \to \pi^{*}$ states, and according to El-Sayed rules, ISC transitions between such states are forbidden. Similarly, the SOCC values for the $S_{Q}$ (2.64 eV, Table 5) and T_5−11_ (2.94 eV) states are zero; moreover, the triplet states have higher energy. Transitions between S_1,3_ and $T_{5-11}$ can be possible after ring deformation. As a result of deformation, the $\pi \to \pi^{*}$ states gain *d* orbital admixture, and the energy barrier can be reached. From the point of view of palladium porphyrin applications, it is important that ISC occurs from the Q state. In substituted and extended porphyrins with a non-planar structure, this problem can be solved. There is experimental evidence that such systems have increased phosphorescence intensity and a shifted Q band position [64,66].

The weak fluorescence that appears in some derivatives (Table 7) indicates that this process may compete with ISC from the Q band. In the case of the Soret state, the situation is different. Below the Soret band several states of the $d\to \pi^{*}$ character are found, which can lead to ISC. For example, the S_3−5_ (3.29–3.41 eV) and T_9−11_ (3.12–3.19 eV) states have large SOCC values and energy difference is not very large, in the range of 0.3 eV. Rapid singlet–triplet and series of IC transitions to the T_1_ state of $\pi \to \pi^{*}$ character occur, followed by deactivation to the ground state by phosphorescence. Similar as in the case of NiPor, ISC from the Soret state to triplet states may be faster than IC to the lower energy singlet states. This is supported by the large energy difference between $S_{Q}$ and higher states (around 0.8 eV) and closely located singlets and triplets at around 3.3 eV with large SOCC values.

#### 3.1.2. NiCorr and PdCorr

**Figure 8:**
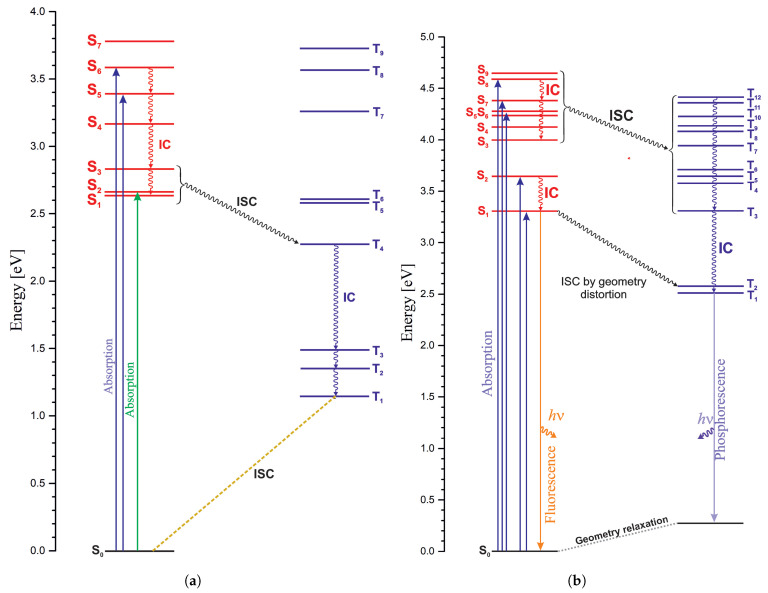
Schematic representation of corrin complexes’ photophysics: (**a**) NiCorr. (**b**) PdCorr.

Figure 8 shows schematic diagrams of the photophysical process paths for nickel corrin and palladium corrin. They were prepared based on the calculated electronic spectra and spin–orbital coupling coefficients (SOCCs). Figure 8a depicts a diagram for NiCorr. The groups of singlet and triplet states for which the SOC coefficients are large are marked with a wavy line.

Although the electronic spectra of nickel corrin species have been measured, the photophysics of these molecules have not been studied. For nickel corrin, there are no time-resolved spectroscopic studies of the photophysical stages. Electronic spectra are known for various derivatives, but no fluorescence or phosphorescence has been observed. Figure 8a shows the proposed process of deactivation of excited states of NiCorr. If we look at the SOCCs gathered in Table 8, one can see that high values for energetically closely lying singlet and triplet states occur for the T_4_-S_1_ and T_4_-S_3_ pairs, with energy difference in the range of 0.36–0.46 eV. Thus, after excitation to the lowest singlet (green arrow in Figure 8) or to singlets with high oscillator strength (blue arrows in Figure 8), a rapid transition occurs via a series of conical intersections (IC) to the S_1_ or S_3_ state and from there ISC to T_4_. Judging by the SOCC values, there is no direct transition from the higher-lying singlet states to triplets. After transition to T_4_, the system proceeds through consecutive IC to T_1_ and from there to the ground state. The calculated energy barrier of this latter process is 3.3 kcal/mol (Figure 3).

The photophysical processes proposed for PdCorr are depicted in Figure 8b. For palladium corrin, upon excitation to the low-lying singlet states (S_1_, S_2_) with high oscillator strengths, ISC to the T_3_ and T_4_ states can occur. PdCorr exhibits both the lowest singlet fluorescence and triplet phosphorescence. From the higher singlet states with high oscillator strengths, ISC processes can occur to T_8_ considering that the SOCCs for T_8_ (4.07 eV) and S_5_ (4.33 eV) have a large value (Table 8) and a relatively small energy difference (about 0.3 eV).

#### 3.1.3. PdPor and PdCorr

Comparison of the photophysical properties of palladium porphyrin (Figure 7b) and palladium corrin (Figure 8b) is also interesting. In PdPor, ISC to the T_1−2_ states is impossible, and similarly, in PdCorr, there is no transition from S_1−2_ to T_1−2_ (as shown by the SOCC values in Table 8); although an S_2_-T_4_ transition is possible judging by the respective SOCC values and close energy of the two states. If excitation to the lowest singlets takes place, ISC to triplet states can occur through ring deformation, which introduces d orbitals into the π orbitals, which affects the SOCC values. If the substituents in the porphyrin ring do not introduce structural distortions, such singlet–triplet transitions may require a barrier, in which case fluorescence is also possible, although weak for both compounds (Table 7). From the higher singlet states, given the large SOCC values and close energies of singlets and triplets, rapid conversion to the triplet state, followed by the transition to T_1_ via the IC series, and phosphorescence are possible. Both complexes show many similarities, but given the intensity of the S_1_ state in PdCorr, fluorescence seems more likely.

### 3.2. Comparison of TDDFT and CASSCF/NEVPT2 Results

#### 3.2.1. NiPor

The electronic spectra for NiPor are presented in Table 2 and Table 9 for TDDFT and NEVPT2 methods, respectively. For NiPor, a comparison of CASSCF/NEVPT2 calculations can begin by comparing the electronic transitions belonging to the Q band and the B band (Soret). These are formed by four π Gouterman orbitals. In NEVPT2, the Q band occurs at 517 nm in very good agreement with the experiment (Table 2). The B band occurs at 387 nm, a wavelength somewhat shorter than the experimental one, but in good agreement with the lower bound of the experimental values in Table 2. In NEVPT2 below the Q band transitions, a series of d→d, $d\to \pi^{*}$, and π→d transitions appear. On the other hand, between the Q and B bands, there is one d→d and two $d\to \pi^{*}$ transitions for the $d_{x^{2}-y^{2}}$ orbital. In TDDFT both Q and B transitions occur at shorter wavelengths than the experimental ones (474 and 325 nm, respectively, compared to the 517–543 and 393–415 experimental values, Table 2). At energies lower than the Q band, three d→d transitions (with π admixture) occur, and between the Q and B transitions, $d\to \pi^{*}$ and π−d transitions appear.

In both methods, the first three d→d triplet transitions have very low energies, 0.23–0.3 eV and 0.36–0.6 eV in NEVPT2 and TDDFT, respectively. This shows an agreement between the methods, which is particularly important from the perspective of photophysical processes. At higher energies, we have $\pi \to \pi^{*}$ and mixed $d\to \pi^{*}$ transitions.

#### 3.2.2. NiCorr

The calculated electronic transitions for NiCorr are gathered in Table 3 for the TDDFT method and Table 10 for the NEVPT2 method. The lowest transitions are d→d, $d\to \pi^{*}$ and π→d transitions. In corrin complexes, *d* and π orbitals mix due to lower symmetry, since the ring is not planar. The first transition with higher intensity is the $\pi \to \pi^{*}$ transition at 364 nm in TDDFT and at 462 nm in NEVPT2. Experimentally, this transition occurs at 440–460 nm (Table 3, depending on the specific form of corrin). It can be seen that TDDFT has a shorter wavelength than the experimental one, while NEVPT2 shows a good agreement. The next high-intensity transition occurs in TDDFT at 284 nm with a $\pi \to \pi^{*}$ character and a small *d* admixture. The equivalent in NEVPT2 occurs at 342 nm. The experimental band occurs at 320–340 nm, in better agreement with the NEVPT2 method. The band that occurs experimentally at around 300 nm appears at 271 nm and 305 nm, corresponding to the TDDFT and NEVPT2, respectively, demonstrating that NEVPT2 gives better agreement.

Generally, singlets in TDDFT are shifted toward higher energies. The same can be observed for triplets. NEVPT2 produces three triplets at very low energies, 0.4–0.6 eV. In TDDFT, the corresponding transitions occur at 1.2–1.5 eV, i.e., at higher energies. In this, NiCorr is different from NiPor where both methods give triplet d→d transitions at very low energy. Generally, the NEVPT2 calculated spectra are in good agreement with the experiment (Table 5), while TDDFT transition energies are shifted to higher energies. However, the order of transitions and their nature are similar in both methods.

#### 3.2.3. PdPor

For PdPor, the calculated spectra are presented in Table 5 and Table 11 for TDDFT and NEVPT2, respectively. The lowest-energy states are the two states belonging to the Q band. They occur at 468 and 507 nm in TDDFT and NEVPT2, respectively. Experimentally, this band is located at 512–527 nm. A better agreement can be observed for NEVPT2. Transitions corresponding to the B band occur at 326 nm in TDDFT and 380 nm in NEVPT2. Again, NEVPT2 provides better agreement with experiment. $d\to \pi^{*}$ transitions occur between the Q and B states in both methods, although they are more numerous in TDDFT. The d→d states occur above the B band, at 290–314 nm in both methods.

Triplet states begin at an energy of approximately 2.1 eV and arise from the $\pi \to \pi^{*}$ states. Starting from 2.94 eV in TDDFT and 3.07 eV in NEVPT2, excited states involving d orbitals begin.

A qualitative agreement of the spectra can be observed between both methods, but NEVPT2 gives a better agreement with the wavelengths of electronic transitions of the experiment, while in the TDDFT method the electronic transitions are shifted towards shorter wavelengths.

#### 3.2.4. PdCorr

The lowest-lying electronic transitions occur at 367 and 343 nm in TDDFT (Table 6) and 382 and 368 nm in NEVPT2 (Table 12). These are the $\pi \to \pi^{*}$ and $d\to \pi^{*}$ transitions. Experimentally, they have been observed at 460 and 430–440 nm. Both methods yield wavelengths that are too short, but NEVPT2 performs slightly better. For PdCorr, there are no experimental measurements for higher electronic states. One can look at the NiCorr spectrum to see what transitions occur there. Experimental bands occur around 320–340 and 300 nm, and around 250–270 nm. In the TDDFT method, there is a transition at 285 nm, which can be compared with the experimental one at around 320 nm. In NEVPT2, there are two transitions with high oscillator strength at 322 and 299 nm, which correspond to the experimental transitions in fairly good agreement. The next higher-energy, high oscillator strength transitions are at 259 and 249 nm in TDDFT, which can be assigned to the experimental band at 240–270 nm. Corresponding transitions occur in NEVPT2 at 258 and 227 nm. All of the above-mentioned transition groups have a large contribution of $\pi \to \pi^{*}$ excitations, with significant contributions from $d\to \pi^{*}$, π→d, and $\pi \to \pi^{*}$. The contribution of $\pi \to \pi^{*}$ transitions contributes to the high deoscillator strength (intensity) of these transitions. A comparison of the TDDFT spectra for NiCorr and PdCorr shows that they appear at similar energies, which is also due to the large contribution of π→π transitions. This justifies the comparison of the calculated PdCorr spectrum to the experimental NiCorr spectrum. For PdCorr, it can be concluded that in NEVPT2 the transitions are shifted towards higher energies, although not as much as in TDDFT. However, qualitatively, the transitions in both spectra determined by both methods show essentially the same character. The lowest triplet transition appears at 2.55 eV in TDDFT and 2.82 eV in NEVPT2. At higher energies, the character of the transitions is similar in both methods, but the energies of TDDFT are higher than those of NEVPT2.

### 3.3. Comparison of Nickel and Palladium Complexes

It is interesting why there is such a difference in photophysical properties between nickel and palladium, given their identical electronic configuration. It can also be noted that MO diagrams for nickel and palladium complexes are very similar. Ake and Gouterman [37] point out that the palladium–porphyrin ligand bond is stronger than that of nickel, since palladium is more covalent. This is a consequence of the larger space extention of the 4d orbitals. Consequently, these compounds are more stable. The high energy of the $d_{x^{2}-y^{2}}$ orbital causes the d→d transitions to have higher energy. This is confirmed by the values of orbital energies gathered Table 13. It shows the HOMO–LUMO energy difference and the energy difference of the occupied and unoccupied orbital $d_{z^{2}}$ and $d_{x2-y2}$, respectively. Although the HOMO–LUMO energy difference is similar for nickel and palladium complexes, the *d* orbital energy difference for nickel is smaller by 0.8 to 0.9 eV. Note that the HOMO and LUMO orbitals are π orbitals in all complexes (Figure 1 and Figure 4). It can be seen that in the palladium complexes, the unoccupied $d_{x^{2}-y^{2}}$ orbital is significantly shifted upwards. This is an antibonding orbital in the metal–ring bond of corrin or porphyrin. Hence, in palladium complexes, transitions involving d orbitals (d→d, $d\to \pi^{*}$, π→d) appear above the Q band.

In addition to low-lying singlet excited states involving *d* orbitals, nickel complexes are also characterized by low-energy triplet states, also with participation of d orbitals. This is visible in both the TDDFT and NEVPT2 calculation results. This property of nickel complexes causes rapid deactivation of excited states via triplet states. If we compare the energy of singlet and triplet excited states, the former always have higher energy. The energy difference between a singlet and a triplet is a double exchange integral formed from the orbitals between which the excitation occurs (Equation (1)) [67].

(1) E(S)−E(T)=2K(a,b),

Exchange integrals can be expressed by Racah parameters [68]:

(2) $$K_{xy,z^{2}}=K_{x^{2}-y^{2},z^{2}}=4B+C$$

In Table 14, Racah parameters for Ni^2+^ and Pd^2+^ cations calculated with AILFT method are gathered.

Although the calculated Racah parameter values seem too large when comparing the energies of singlet and triplet states, they clearly indicate differences between the nickel and palladium complexes. The nickel parameters are significantly larger, resulting in a lower energy of the triplet states relative to the singlet states. The exchange integral K($d_{x^{2}-y^{2}},d_{z2}$) is larger for nickel than for palladium, which is also a consequence of the more spatially compact structure of the 3d orbitals than the 4d orbitals.

## 4. Materials and Methods

All calculations were performed with the use of the ORCA v. 6 package [69,70]. The DFT [71,72] and TDDFT [73] methods were applied in the calculations with the use of the hybrid PBE0 functional [74,75]. The def2-TZVP basis function [76] was applied with effective core potential (ECP) for palladium. In addition, the RIJCOSX approximation [77] for the Coulomb and exchange was utilized. The noncovalent interactions were described by D3BJ dispersion corrections [78]. The full optimization of the complexes structures was carried out with the DFT method. The Tamm–Dancoff approximation was used in the calculations [79]. It is known for giving good triplet states. For the fluorescence the first singlet excited state was optimized with the TDDFT method, while for the phosphorescence the lowest triplet state was optimized with the unrestricted DFT method. All calculations were performed without symmetry. The minimum energy crossing point (MECP) [80] between the lowest triplet state T_1_ and the ground state S_0_ was optomized for all complexes. To get a starting point for the MECP optimization one N-M-N angle was bent until T_1_ and S_0_ crossed. The Racah parameters were calculated with the *ab initio* Ligand Field Theory (AI-LFT) [81] as implemented in the ORCA program.

### 4.1. Density Functional

The PBE0 functional is known as one which adequately depicts the electronic structure of excited states of transition metal complexes [82,83]. In Table S10 the dependence of the excitation energy on the functional type for the $S_{Q}$ and $S_{B}$ states and the three lowest triplets for nickel porphyrin and palladium porphyrin complexes are presented. For hybrid functionals, the contribution of the HF exchange energy is shown. The transition energies calculated using the TDDFT method for nickel porphyrin and palladium porphyrin were compared to the experiment and with those obtained using the CASSCF/NEVPT2 method. Comparison with the NEVPT2 results is important for triplets, where experimental data are lacking. For nickel porphyrin, gradient functionals yield relatively good energies for the Q and B states, but the energies of the triplet states are much too high, by about 1 eV. Long-range hybrid functionals yield worse results for the Q and B states than hybrid functionals, with the latter giving especially worse results for the B state. For palladium porphyrin, gradient functionals are again better at reproducing the energies of the Q and B states; the remaining functionals behave similarly as for nickel porphyrin. Triplets have much higher energy than in nickel porphyrin, and all functionals produce quite similar results in this case. From these results it can be concluded that PBE0 provides a balanced description of singlet and triplet states.

### 4.2. Solvent Model

The continuous solvent model CPCM was used [84] with methanol as a solvent. The experimental data concern various solvents. The spectra of nickel corrin and palladium corrin were measured in methanol, while for nickel porphyrin and palladium porphyrin solvents such as dichloromethane and acetonitrile were used. However, from a calculation methodology perspective, we had to choose a single, specific solvent. Therefore, we chose methanol as a representative with a relatively average dielectric constant. In Table S9, the dependence of the excitation energies on the model and solvent type for the Q and B states and the three lowest triplet states for nickel porphyrin and palladium porphyrin complexes are displayed. In Figure S1, the simulated spectra for the NiPor model complex calculations with use CPCM solvation model and two different solvents are depicted.

The test results clearly show that, overall, neither the method (CPCM and SMD [84]) nor the specific solvent have a significant impact on the results. States in the Q band are practically completely insensitive to solvent influence, at least from the perspective of continuous models. A slightly greater influence of the solvent and its nature can be observed for the $\pi \to \pi^{*}$ states in the Soret band. A similarly small influence applies to the lowest triplet states, especially in the case of PdPor. This is not significant from the perspective of photophysical interpretation. Even if there are slight shifts in these states, the overall picture of the photophysical processes will not be significantly different for different solvents. To maintain consistency in our results, we used the same selected solvent for the corrin complexes.

### 4.3. Spin–Orbit Interaction

To estimate the interactions between singlet and triplet electronic states obtained from TDDFT and determine the values of spin–orbit coupling (SOC) integrals, the formalism of the Quasi-Degenerate Perturbation Theory (QDPT) [85,86] was used with a full electron basis function, ZORA-def2-TZVP [87], for the palladium atom. The geometry was fully optimised in ZORA-def2-TZVP/SARC-ZORA-TZVP basis set. In Table S1, the vertical singlet electronic transitions for the PdPor complex based on the TDDFT/PBE0/ZORA-def2-TZVP/SARC-ZORA-TZVP are shown.

The rate constant for non-radiative ISC ($k_{ISC}$) can be obtained from the Fermi Golden Rule according the formula [88,89]:

(3) $$k_{ISC}^{nm}=\frac{2\pi }{\hbar }\langle S_{n}|{\hat{H}}_{SO}{|T_{m}\rangle }^{2}\times \mathrm{FCWD}.$$

FCWD is the Frank–Condon weighted density of states which can be approximately determined from the Marcus theory:

(4) $$\mathrm{FCWD}\approx \frac{1}{\sqrt{4\pi \lambda \mathrm{RT}}}exp\left(-\frac{{\left(\Delta E+\lambda \right)}^{2}}{4\pi \lambda \mathrm{RT}}\right),$$

where ΔE is the barrier from $S_{n}$ to $T_{m}$ and λ is the reorganization energy. The Marcus theory approximation does not always give good results [90]. Close energy states have a small ΔE barrier. Possible intersystem crossing pathways from singlet excited state to higher triplet states and finally tle lowest triplet were proposed on the basis of the calculated SOCC and the singlet–triplet energy difference [57].

### 4.4. CASSCF/NEVPT2 Calculations

The electronic spectra of the studied complexes were also calculated by the CASSCF/NEVPT2 method [91,92,93] with the use of the def2-TZVP basis set on the DFT optimized geometry.

The active space for the corrin complexes consisted of 12 electrons and 12 orbitals (CASSCF(12,12)) (see Figures S3 and S5). This space contains four Goutermann π orbitals, 3d orbitals, including three doubly occupied ones: $3d_{z^{2}}$, $3d_{xz}$, $3d_{yz}$, and one unoccupied one, i.e., $3d_{xy}$. The $4d_{z^{2}}$, $4d_{xz}$, and $4d_{yz}$ orbitals were added as correlation orbitals to the doubly occupied ones, and an orbital formed from lone electron pairs of nitrogen atoms with the same symmetry as $3d_{xy}$ (denoted as $n_{N}$) to the unoccupied one. The ${3d}_{xy}$ and $n_{N}$ orbitals form a metal–ring coordination bond. The addition of 4d orbitals is important due to the so-called double-d-shell effect [94] which is crucial for describing bonding and spectra in transition metal compounds. The active space for porphyrin complexes contains 13 orbitals and 14 electrons, designated CASSCF(14,13) (Figures S2 and S4). It additionally includes an occupied $3d_{x^{2}-y^{2}}$ orbital.

It is important to determine the appropriate number of excited states in CASSCF/NEVPT2 calculations. Too few states prevent the low-lying states in NEVPT2 from appearing, due to the different effects of dynamical correlation on different states. Thus, the order of states changes dramatically from CASSCF to NEVPT2. This can be seen in Table S7 (Supplementary Materials), where for NiPor, states 1 and 2 in NEVPT2 are states 29 and 28 in CASSCF, respectively. For NiPor, PdPor, NiCorr, and PdCorr, 50, 40, 35, and 35 singlet and triplet states were determined, respectively.

### 4.5. Model Geometries

**Figure 9:**
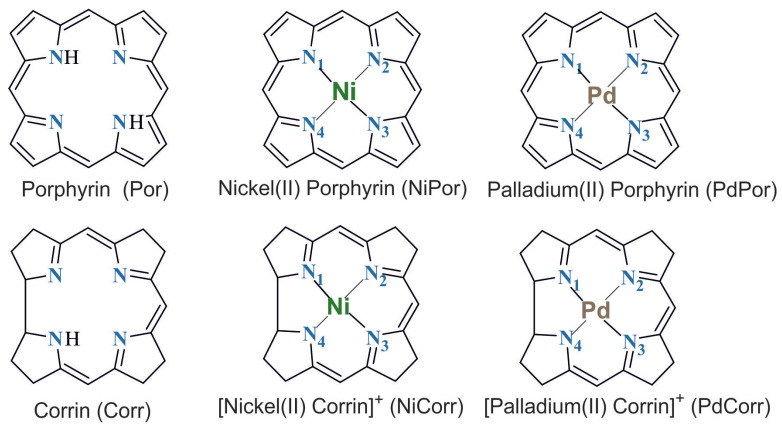
Structure of corrin and porphyrin complexes with the atom numbering and colors used.

The structural formulas of the model complexes that are the subject of this work, as well as the free ligands, porphyrin and corrin, are shown in Figure 9. Porphyrin has two protons on its nitrogen atoms, while corrin has one. Accordingly, the porphyrinate anion has a charge of −2 and the corrinate anion has a charge of −1. The nickel and palladium ions are in the second oxidation state, so the porphyrin complex is neutral and the corrin complex has a charge of +1.

## 5. Conclusions

The nickel(II) and palladium(II) model complexes with the porphyrin and corrin ligands were computationally studied with using DFT/TDDFT and CASSCF/NEVPT2 levels of theory. According to the obtained results, the observed experimental differences in photophysical properties have their origin in the complex energetic redistribution of singlet and triplet excited states interacting with each other in the intersystem crossing process. In the case of nickel(II) porphyrin (NiPor) and nickel(II) corrin (NiCorr) complexes, virtually no emission is observed in experimental measurements. Thus, after occupation of a singlet electronic state in absorption and a rapid transition to the triplet state, a very fast transition to the ground state follows. This situation is favored by the low-lying triplet states in these complexes, as shown by the results of TDDFT and NEVPT2 calculations. For NiPor the singlet Q state undergoes rapid internal conversion to lower-lying states involving d orbitals of nickel, from which a transition via ISC occurs to low-energy triplet states. A similar mechanism is predicted for the quenching of excited states in the Soret band. The excited $\pi \to \pi^{*}$ states thermally relax to lower d/π mixed states, after which these states undergo intersystem crossing with triplet states. Next, internal conversion within triplet states ultimately leads to quenching of the excitation energy via T_1_/S_0_ ISC. For NiCorr, the computational results indicate that the mechanism of excited state quenching involving triplet states is similar to that of the porphyrin complex. However, in this case, all absorptively excited singlet states undergo internal conversion to low-lying states according to Kasha’s rule. Due to the mixed d/π nature of these states, it is possible to efficiently transition the ISC to higher triplet states, from which the lowest triplet state is occupied as a result of relaxation. This state then undergoes radiationless deactivation to the ground state. In tetrapyrrole palladium(II) complexes, the mechanisms of internal conversion of excited states as well as the interaction of singlet and triplet states are very similar to those considered in the case of nickel(II) complexes. However, in palladium complexes, photophysically active low-lying triplet states have a much higher energy relative to the ground state compared to the energy distribution of triplet states in nickel complexes. In general, radiationless quenching of triplet states is unlikely here, so the main photophysical mechanism leading to deactivation of the complexes in this case is phosphorescence. Alternatively, low-lying triplet states may be deactivated by intra- or intermolecular energy transfer.

## Figures and Tables

**Table 2:** Electronic transitions of NiPor calculated by TDDFT/PBE0/def2-TZVP and CPCM/methanol solvent model.

No	E(eV)	λ(nm)	*f*	Orbitals	Weight	$\lambda_{\exp}$
Singlet transitions						
1	1.95	633.6	0.0000	$H-4\left(d_{z^{2}}\right)\to L+2\left(d_{x^{2}-y^{2}}\right)$	0.976	
2	2.04	606.3	0.0000	$H-2\left(\pi +d_{xz}\right)\to L+2\left(d_{x^{2}-y^{2}}\right)$	0.651	
3	2.04	606.3	0.0000	$H-9\left(d+\pi \right)\to L+2\left(d_{x^{2}-y^{2}}\right)$	0.320	
				$H-3\left(\pi +d_{yz}\right)\to L+2\left(d_{x^{2}-y^{2}}\right)$	0.651	
4	2.49	496.3	0.0000	$H-11\left(d_{xy}\right)\to L+2\left(d_{x^{2}-y^{2}}\right)$	0.951	
5	2.61	474.1	0.0119	$H-1\left(\pi_{1}\right)\to L\left(\pi_{3}^{*}\right)$	0.447	525 ^a^, 530 ^b^, 522 ^c^
				$H\left(\pi_{2}\right)\to L+1\left(\pi_{4}^{*}\right)$	0.540	516 ^d^, 543 ^e^, 527 ^f^, 517 ^g^
6	2.61	474.1	0.0119	$H-1\left(\pi_{1}\right)\to L+1\left(\pi_{4}^{*}\right)$	0.447	
				$H\left(\pi_{2}\right)\to L\left(\pi_{3}^{*}\right)$	0.540	
7	3.14	393.8	0.0000	$H-1\left(\pi_{1}\right)\to L+2\left(d_{x^{2}-y^{2}}\right)$	0.985	
8	3.16	391.5	0.0000	$H\left(\pi_{2}\right)\to L+2\left(d_{x^{2}-y^{2}}\right)$	0.999	
9	3.42	362.1	0.0000	$H-3\left(\pi +d_{yz}\right)\to L\left(\pi_{3}^{*}\right)$	0.489	
				$H-2\left(\pi +d_{xz}\right)\to L+1\left(\pi_{4}^{*}\right)$	0.491	
10	3.48	356.2	0.0000	$H-3\left(\pi +d_{yz}\right)\to L\left(\pi_{3}^{*}\right)$	0.492	
				$H-2\left(\pi +d_{xz}\right)\to L+1\left(\pi_{4}^{*}\right)$	0.491	
11	3.57	347.0	0.0000	$H-3\left(\pi +d_{yz}\right)\to L+1\left(\pi_{4}^{*}\right)$	0.478	
				$H-2\left(\pi +d_{xz}\right)\to L\left(\pi_{3}^{*}\right)$	0.479	
12	3.65	339.4	0.0000	$H-4\left(d_{z^{2}}\right)\to L\left(\pi_{3}^{*}\right)$	0.995	
13	3.65	339.4	0.0000	$H-4\left(d_{z^{2}}\right)\to L+1\left(\pi_{4}^{*}\right)$	0.995	
14	3.81	325.1	1.7872	$H-1\left(\pi_{1}\right)\to L\left(\pi_{3}^{*}\right)$	0.464	415 ^a^, 415 ^b^, 414 ^c^, 393 ^d^
				$H\left(\pi_{2}\right)\to L+1\left(\pi_{4}^{*}\right)$	0.396	393 ^e^, 415 ^f^, 415 ^g^, 392 ^h^
15	3.81	325.1	1.7879	$H-1\left(\pi_{1}\right)\to L+1\left(\pi_{4}^{*}\right)$	0.464	
				$H\left(\pi_{2}\right)\to L\left(\pi_{3}^{*}\right)$	0.396	
16	3.81	325.0	0.0005	$H-3\left(\pi +d_{yz}\right)\to L+1\left(\pi_{4}^{*}\right)$	0.477	
				$H-2\left(\pi +d_{xz}\right)\to L\left(\pi_{3}^{*}\right)$	0.477	
Triplet transitions						
1	0.353	3505.5		$H-4\left(d_{z^{2}}\right)\to L+2\left(d_{x^{2}-y^{2}}\right)$	0.983	
2	0.609	2034.1		$H-9\left(d+\pi \right)\to L+2\left(d_{x^{2}-y^{2}}\right)$	0.297	
				$H-3\left(\pi +d_{yz}\right)\to L+2\left(d_{x^{2}-y^{2}}\right)$	0.428	
3	0.609	2034.1		$H-10\left(d+\pi \right)\to L+2\left(d_{x^{2}-y^{2}}\right)$	0.297	
				$H-2\left(\pi +d_{xz}\right)\to L+2\left(d_{x^{2}-y^{2}}\right)$	0.428	
4	1.553	797.9		$H-11\left(d_{xy}\right)\to L+2\left(d_{x^{2}-y^{2}}\right)$	0.943	
5	2.076	597.1		$H-1\left(\pi_{1}\right)\to L+1\left(\pi_{4}^{*}\right)$	0.440	
				$H\left(\pi_{2}\right)\to L\left(\pi_{3}^{*}\right)$	0.527	
6	2.076	597.1		$H-1\left(\pi_{1}\right)\to L\left(\pi_{3}^{*}\right)$	0.440	
				$H\left(\pi_{2}\right)\to L+1\left(\pi_{4}^{*}\right)$	0.527	
7	2.213	560.0		$H\left(\pi_{1}\right)\to L+1\left(\pi_{4}^{*}\right)$	0.534	
				$H\left(\pi_{2}\right)\to L\left(\pi_{3}^{*}\right)$	0.458	
8	3.066	404.4		$H\left(\pi_{1}\right)\to L+2\left(d_{x^{2}-y^{2}}\right)$	0.975	
9	3.097	400.2		$H-3\left(\pi +d_{yz}\right)\to L\left(\pi_{3}^{*}\right)$	0.465	
				$H\left(\pi_{2}\right)\to L+1\left(\pi_{4}^{*}\right)$	0.466	
10	3.146	394.0		$H-3\left(\pi +d_{yz}\right)\to L\left(\pi_{3}^{*}\right)$	0.466	
				$H\left(\pi_{2}\right)\to L+1\left(\pi_{4}^{*}\right)$	0.466	
11	3.149	393.7		$H\left(\pi_{2}\right)\to L+2\left(d_{x^{2}-y^{2}}\right)$	0.999	
12	3.192	388.3		$H-3\left(\pi +d_{yz}\right)\to L+1\left(\pi_{4}^{*}\right)$	0.478	
				$H-2\left(\pi +d_{xz}\right)\to L\left(\pi_{3}^{*}\right)$	0.481	
13	3.246	381.9		$H-3\left(\pi +d_{yz}\right)\to L+1\left(\pi_{4}^{*}\right)$	0.477	
				$H-2\left(\pi +d_{xz}\right)\to L\left(\pi_{3}^{*}\right)$	0.474	
14	3.583	346.0		$H-5\left(\pi \right)\to L\left(\pi_{3}^{*}\right)$	0.757	
15	3.583	346.0		$H-5\left(\pi \right)\to L+1\left(\pi_{4}^{*}\right)$	0.757	

^a^ Ref. [21], NiTPP; ^b^ Ref. [39], NiTPP; ^c^ Ref. [29], NiTMP; ^d^ Ref. [30], NiOTP; ^e^ Ref. [35], NiOEP; ^f^ Ref. [36], NiTMP; ^g^ Ref. [37] NiTPP; ^h^ Ref. [37] NiOEP

**Table 3:** Electronic transitions of NiCorr calculated by TDDFT/PBE0/def2-TZVP and CPCM/methanol solvent model.

No	E(eV)	λ(nm)	*f*	Orbitals	Weight	$\lambda_{\exp}$
Singlet transitions						
1	2.664	465.4	0.0000	$H-4\left(d+\pi \right)\to L+2\left(d_{x^{2}-y^{2}}\right)$	0.319	
				$H-1\left(\pi_{1}+d\right)\to L+2\left(d_{x^{2}-y^{2}}\right)$	0.400	
2	2.693	460.4	0.0010	$H-3(d_{z^{2}})\to L+2\left(d_{x^{2}-y^{2}}\right)$	0.746	
3	2.863	433.1	0.0001	$H-2\left(\pi +d_{xz}\right)\to L+2\left(d_{x^{2}-y^{2}}\right)$	0.528	
4	3.195	388.0	0.0030	$H-7\left(d_{xy}\right)\to L+2\left(d_{x^{2}-y^{2}}\right)$	0.567	
5	3.402	364.4	0.2503	$H\left(\pi_{2}\right)\to L\left(\pi_{3}^{*}\right)$	0.876	460 ^e^, 450 ^b^, 454 ^c^
6	3.588	345.5	0.1723	$H-1\left(\pi_{1}+d\right)\to L\left(\pi_{3}^{*}\right)$	0.870	416 ^c^, 427 ^b^, 428 ^d^, 435 ^a^
7	3.781	327.9	0.0013	$H-3(d_{z^{2}})\to L\left(\pi_{3}^{*}\right)$	0.989	
8	3.977	311.8	0.0058	$H-2\left(\pi +d_{xz}\right)\to L\left(\pi_{3}^{*}\right)$	0.790	
9	4.004	309.6	0.0347	$H\left(\pi_{2}\right)\to L+1\left(\pi_{4}^{*}+d\right)$	0.270	
				$H\left(\pi_{2}\right)\to L+2\left(d_{x^{2}-y^{2}}\right)$	0.687	
10	4.365	284.1	0.2440	$H-1\left(\pi_{1}+d\right)\to L+1\left(\pi_{4}^{*}+d\right)$	0.591	317 ^d^, 319 ^a^, 320 ^e^, 340 ^e^
11	4.575	271.0	0.4652	$H\left(\pi_{2}\right)\to L+1\left(\pi_{4}^{*}+d\right)$	0.543	294 ^c^, 304 ^d^, 306 ^d^ 312 ^c^
				$H\left(\pi_{2}\right)\to L+2\left(d_{x^{2}-y^{2}}\right)$	0.262	
12	4.638	267.3	0.0024	$H-3(d_{z^{2}})\to L+1\left(\pi_{4}^{*}+d\right)$	0.638	
13	4.729	262.2	0.0011	$H-4\left(d_{yz}+\pi \right)\to L+2\left(d_{x^{2}-y^{2}}\right)$	0.264	
				$H-1\left(\pi_{1}+d\right)\to L+2\left(d_{x^{2}-y^{2}}\right)$	0.342	
14	4.833	256.5	0.2251	$H-4\left(d_{yz}+\pi \right)\to L\left(\pi_{3}^{*}\right)$	0.755	244 ^d^, 245 ^a^, 277 ^d^, 266 ^d^
15	5.122	242.1	0.0244	$H-5\left(\pi +d\right)\to L\left(\pi_{3}^{*}\right)$	0.670	
16	5.154	240.6	0.0180	$H-1\left(\pi_{1}+d\right)\to L+3\left(\pi \right)$	0.721	
Triplet transitions						
1	1.182	1049.2	0.0000	$H-3\left(d_{z^{2}}\right)\to L+2\left(d_{x^{2}-y^{2}}\right)$	0.786	
2	1.385	895.4	0.0000	$H-4\left(d_{yz}+\pi \right)\to L+2\left(d_{x^{2}-y^{2}}\right)$	0.439	
				$H-1\left(\pi_{1}+d\right)\to L+2\left(d_{x^{2}-y^{2}}\right)$	0.308	
3	1.525	812.9	0.0000	$H-2\left(\pi +d_{xz}\right)\to L+2\left(d_{x^{2}-y^{2}}\right)$	0.494	
4	2.306	537.6	0.0000	$H-7\left(d_{xy}\right)\to L+2\left(d_{x^{2}-y^{2}}\right)$	0.597	
5	2.579	480.7	0.0000	$H-1\left(\pi_{1}+d\right)\to L\left(\pi_{3}^{*}\right)$	0.903	
6	2.617	473.8	0.0000	$H\left(\pi_{2}\right)\to L\left(\pi_{3}^{*}\right)$	0.912	
7	3.266	379.6	0.0000	$H-2\left(\pi +d_{xz}\right)\to L\left(\pi_{3}^{*}\right)$	0.430	
				$H-1\left(\pi_{1}+d\right)\to L+1\left(\pi_{4}^{*}+d\right)$	0.341	
8	3.577	346.6	0.0000	$H\left(\pi_{2}\right)\to L+1\left(\pi_{4}^{*}+d\right)$	0.775	
9	3.728	332.6	0.0000	$H-3\left(d_{z^{2}}\right)\to L\left(\pi_{3}^{*}\right)$	0.985	
10	3.923	316.1	0.0000	$H\left(\pi_{2}\right)\to L+2\left(d_{x^{2}-y^{2}}\right)$	0.888	
11	3.939	314.8	0.0000	$H-2\left(\pi +d_{xz}\right)\to L\left(\pi_{3}^{*}\right)$	0.540	
				$H-1\left(\pi_{1}+d\right)\to L+1\left(\pi_{4}^{*}+d\right)$	0.320	
12	4.227	293.3	0.0000	$H-2\left(\pi +d_{xz}\right)\to L+1\left(\pi_{4}^{*}+d\right)$	0.457	
				$H-1\left(\pi_{1}+d\right)\to L+3\left(\pi^{*}\right)$	0.203	
13	4.337	285.9	0.0000	$H-5\left(\pi +d\right)\to L\left(\pi_{3}^{*}\right)$	0.483	

^a^ Ref. [14] corrin perchlorate.; ^b^ Ref. [15]; ^c^ Ref. [16]; ^d^ Ref. [19]; ^e^ Ref. [5], Nibl.

**Table 4:** Selected geometry parameters of PdCorr and PdPor. Bond lengths in Å, angles in degrees.

Parameter	PdCorr		PdPor	
	Calc.	Expt. ^a^	Calc.	Expt. ^b^
Pd-N_1_	1.931	2.025	2.017	2.017
Pd-N_2_	1.971	2.009	2.017	2.011
Pd-N_3_	1.971	2.018	2.017	2.017
Pd-N_4_	1.931	2.005	2.017	2.011
N_1_-Pd-N_2_	92.2	89.9	90.0	90.1
N_2_-Pd-N_3_	94.4	92.0	90.0	89.9
N_3_-Pd-N_4_	92.2	89.0	90.0	90.1
N_4_-Pd-N_1_	81.4	92.2	90.0	89.9
N_1_-Pd-N_3_	172.7	167.4	180.0	180.0
N_2_-Pd-N_4_	172.7	166.3	180.0	180.0
N_1_-N_2_-N_3_-N_4_	−4.3	−18.5	0.0	0.0
N_1_-N_2_-N_3_-Pd	−2.2	−8.1	0.0	0.0

^a^ Ref. [62], ^b^ Ref. [63].

**Table 5:** Electronic transitions of PdPor calculated by TDDFT/PBE0/def2-TZVP and CPCM/methanol solvent model.

No	E(eV)	λ(nm)	*f*	Orbitals	Weight	$\lambda_{\exp}$
Singlet transitions						
1	2.64	468.6	0.0104740	$\pi_{1}\to \pi_{3}^{*}$	0.401	512 ^a^,514 ^c^, 514 ^e^, 519 ^d^
				$\pi_{2}\to \pi_{4}^{*}$	0.471	523 ^e^, 524 ^f^, 527 ^d^
2	2.64	468.6	0.0104772	$\pi_{1}\to \pi_{4}^{*}$	0.401	
				$\pi_{2}\to \pi_{3}^{*}$	0.471	
3	3.29	375.9	0.0000000	$d_{yz}+\pi \to \pi_{3}^{*}$	0.491	
				$d_{yz}+\pi \to \pi_{4}^{*}$	0.491	
4	3.35	369.5	0.0000000	$d_{yz}+\pi \to \pi_{3}^{*}$	0.492	
				$d_{yz}+\pi \to \pi_{4}^{*}$	0.492	
5	3.41	362.8	0.0000000	$d_{yz}+\pi \to \pi_{4}^{*}$	0.458	
				$d_{yz}+\pi \to \pi_{3}^{*}$	0.458	
6	3.72	333.0	0.0000000	$d_{yz}+\pi \to \pi_{4}^{*}$	0.484	
				$d_{yz}+\pi \to \pi_{3}^{*}$	0.483	
7	3.76	329.2	0.0018097	$d_{z^{2}}\to \pi_{3}^{*}$	0.986	
8	3.76	329.2	0.0018113	$d_{z^{2}}\to \pi_{4}^{*}$	0.986	
9	3.79	326.4	1.7061422	$\pi_{1}\to \pi_{3}^{*}$	0.450	394 ^a^, 394 ^e^, 406 ^d^, 415 ^d^
				$\pi_{2}\to \pi_{4}^{*}$	0.399	416 ^c^, 417 ^e^,417 ^f^, 418 ^b^
10	3.79	326.4	1.7062120	$\pi_{1}\to \pi_{4}^{*}$	0.450	
				$\pi_{2}\to \pi_{3}^{*}$	0.399	
11	3.84	322.5	0.0000003	$\pi_{1}\to d_{x^{2}-y^{2}}$	0.987	
12	3.87	320.2	0.0000000	$\pi_{4}\to d_{x^{2}-y^{2}}$	0.998	
13	3.94	314.1	0.0000063	$d_{yz}+\pi \to d_{x^{2}-y^{2}}$	0.863	
14	3.94	314.1	0.0000068	$d_{yz}+\pi \to d_{x^{2}-y^{2}}$	0.863	
15	4.07	304.3	0.0000000	$d_{z^{2}}\to d_{x^{2}-y^{2}}$	0.916	
Triplet transitions						
1	2.09	591.4	0.00000	$\pi_{1}\to \pi_{3}^{*}$	0.474	
				$\pi_{2}\to \pi_{4}^{*}$	0.250	
2	2.09	591.4	0.00000	$\pi_{1}\to \pi_{4}^{*}$	0.474	
				$\pi_{2}\to \pi_{3}^{*}$	0.250	
3	2.24	553.4	0.00000	$\pi_{1}\to \pi_{4}^{*}$	0.345	
				$\pi_{2}\to \pi_{3}^{*}$	0.648	
4	2.24	553.4	0.00000	$\pi_{1}\to \pi_{3}^{*}$	0.345	
				$\pi_{2}\to \pi_{4}^{*}$	0.648	
5	2.94	420.5	0.00000	$d_{yz}+\pi \to \pi_{3}^{*}$	0.464	
				$d_{xz}+\pi \to \pi_{4}^{*}$	0.464	
6	2.99	413.6	0.00000	$d_{xz}+\pi \to \pi_{4}^{*}$	0.464	
				$d_{xz}+\pi \to \pi_{4}^{*}$	0.467	
7	3.06	404.2	0.00000	$d_{yz}+\pi \to \pi_{4}^{*}$	0.479	
				$d_{xz}+\pi \to \pi_{3}^{*}$	0.481	
8	3.11	398.4	0.00000	$d_{z^{2}}\to d_{x^{2}-y^{2}}$	0.545	
				$d_{yz}+\pi \to \pi_{4}^{*}$	0.213	
				$d_{xz}+\pi \to \pi_{4}^{*}$	0.213	
9	3.11	397.4	0.00000	$d_{z^{2}}\to d_{x^{2}-y^{2}}$	0.439	
				$d_{yz}+\pi \to \pi_{4}^{*}$	0.263	
				$d_{xz}+\pi \to \pi_{3}^{*}$	0.263	
10	3.18	388.9	0.00000	$d_{xz}+\pi \to d_{x^{2}-y^{2}}$	0.751	
11	3.18	388.9	0.00000	$d_{yz}+\pi \to d_{x^{2}-y^{2}}$	0.751	
12	3.59	345.1	0.00000	$\pi_{2}\to \pi_{3}^{*}$	0.662	

^a^ Ref. [43] PdOEP; ^b^ Ref. [41] PdTPP; ^c^ Ref. [44] PdTPP; ^d^ Ref. [19];^e^ Ref. [64] PdOEP, PdTPP; ^f^ Ref. [50], PdTPP.

**Table 6:** Electronic transitions of PdCorr calculated by TDDFT/PBE0/def2-TZVP and CPCM/methanol solvent model.

No	E(eV)	λ(nm)	*f*	Orbitals	Weight	$\lambda_{\exp}$
Singlet transitions						
1	3.376	367.2	0.2597	$H\left(\pi_{2}\right)\to L\left(\pi_{3}^{*}\right)$	0.906	460 ^a^, 461 ^b^
2	3.616	342.9	0.2067	$H-1\left(\pi_{1}+d_{yz}\right)\to L\left(\pi_{3}^{*}\right)$	0.890	430 ^a^, 441 ^b^
3	3.980	311.5	0.0000	$H-3\left(d_{z^{2}}\right)\to L\left(\pi_{3}^{*}\right)$	0.994	
4	4.093	302.9	0.0057	$H-2\left(\pi +d_{xz}\right)\to L\left(\pi_{3}^{*}\right)$	0.711	
				$H-1\left(\pi_{1}+d_{yz}\right)\to L+1\left(\pi_{4}^{*}\right)$	0.235	
5	4.338	285.8	0.1706	$H-1\left(\pi_{1}+d_{yz}\right)\to L+1\left(\pi_{4}^{*}\right)$	0.370	
				$H-1\left(\pi_{1}+d_{yz}\right)\to L+3\left(d_{x^{2}-y^{2}}\right)$	0.291	
6	4.509	275.0	0.1052	$H-1\left(\pi_{1}+d_{yz}\right)\to L+3\left(d_{x^{2}-y^{2}}\right)$	0.578	
7	4.533	273.5	0.4470	$H\left(\pi_{2}\right)\to L+1\left(\pi_{4}^{*}\right)$	0.689	
8	4.768	260.0	0.0054	$H-3\left(d_{z^{2}}\right)\to L+1\left(\pi_{4}^{*}\right)$	0.313	
				$H-3\left(d_{z^{2}}\right)\to L+3\left(d_{x^{2}-y^{2}}\right)$	0.441	
9	4.776	259.6	0.1415	$H\left(\pi_{2}\right)\to L+3\left(d_{x^{2}-y^{2}}\right)$	0.782	
10	4.978	249.0	0.0119	$H-2\left(\pi +d_{xz}\right)\to L+3\left(d_{x^{2}-y^{2}}\right)$	0.615	
11	4.996	248.2	0.0005	$H-3\left(d_{z^{2}}\right)\to L+1\left(\pi_{4}^{*}\right)$	0.653	
				$H-3\left(d_{z^{2}}\right)\to L+3\left(d_{x^{2}-y^{2}}\right)$	0.234	
12	5.074	244.3	0.0800	$H-6\left(d_{xy}\right)\to L\left(\pi_{3}^{*}\right)$	0.316	
				$H-4\left(\pi +d\right)\to L\left(\pi_{3}^{*}\right)$	0.514	
13	5.153	240.6	0.0772	$H-1\left(\pi_{1}+d_{yz}\right)\to L+2\left(\pi^{*}\right)$	0.845	
14	5.251	236.1	0.0034	$H-5\left(\pi +d\right)\to L\left(\pi_{3}^{*}\right)$	0.593	
Triplet transitions						
1	2.553	485.6	0.0000	$H-1\left(\pi_{1}+d_{yz}\right)\to L\left(\pi_{3}^{*}\right)$	0.9088	
2	2.582	480.1	0.0000	$H\left(\pi_{2}\right)\to L\left(\pi_{3}^{*}\right)$	0.9163	
3	3.291	376.7	0.0000	$H-2\left(\pi +d_{xz}\right)\to L\left(\pi_{3}^{*}\right)$	0.4526	
				$H-1\left(\pi_{1}+d_{yz}\right)\to L+1\left(\pi_{4}^{*}\right)$	0.3795	
4	3.621	342.4	0.0000	$H\left(\pi_{2}\right)\to L+1\left(\pi_{4}^{*}\right)$	0.7816	
5	3.855	321.6	0.0000	$H-1\left(\pi_{1}+d_{yz}\right)\to L+3\left(d_{x^{2}-y^{2}}\right)$	0.5608	
6	3.926	315.8	0.0000	$H-3\left(d_{z^{2}}\right)\to L\left(\pi_{3}^{*}\right)$	0.9176	
7	3.930	315.5	0.0000	$H-3\left(d_{z^{2}}\right)\to L+3\left(d_{x^{2}-y^{2}}\right)$	0.7699	
8	4.071	304.5	0.0000	$H-2\left(\pi +d_{xz}\right)\to L\left(\pi_{3}^{*}\right)$	0.4324	
				$H-1\left(\pi_{1}+d_{yz}\right)\to L+1\left(\pi_{4}^{*}\right)$	0.3198	
9	4.198	295.3	0.0000	$H-2\left(\pi +d_{xz}\right)\to L+1\left(\pi_{4}^{*}\right)$	0.2940	
				$H-2\left(\pi +d_{xz}\right)\to L+3\left(d_{x^{2}-y^{2}}\right)$	0.3055	
10	4.334	282.9	0.0000	$H-2\left(\pi +d_{xz}\right)\to L+3\left(d_{x^{2}-y^{2}}\right)$	0.3433	
				$H-1\left(\pi_{1}+d_{yz}\right)\to L+2\left(\pi^{*}\right)$	0.2488	
11	4.382	270.8	0.0000	$H-5\left(\pi +d\right)\to L\left(\pi_{3}^{*}\right)$	0.3007	
				$H\left(\pi_{2}\right)\to L+2\left(\pi^{*}\right)$	0.4203	
12	4.578	264.3	0.0000	$H\left(\pi_{2}\right)\to L+3\left(d_{x^{2}-y^{2}}\right)$	0.7792	
13	4.691	260.7	0.0000	$H-4\left(\pi +d\right)\to L\left(\pi_{3}^{*}\right)$	0.6647	
14	4.756	254.7	0.0000	$H-5\left(\pi +d\right)\to L\left(\pi_{3}^{*}\right)$	0.5090	
				$H\left(\pi_{2}\right)\to L+2\left(\pi^{*}\right)$	0.3927	
15	4.868	253.5	0.0000	$H-3\left(d_{z^{2}}\right)\to L+1\left(\pi_{4}^{*}\right)$	0.8951	
16	4.890	252.5	0.0000	$H-6\left(d_{xy}\right)\to L+3\left(d_{x^{2}-y^{2}}\right)$	0.6370	

^a^ Ref. [15]; ^b^ Ref. [17].

**Table 7:** Emission of nickel and palladium complexes calculated by TDDFT/PBE0/def2-TZVP and CPCM/methanol solvent model.

Molecule	Emission	$\lambda_{\mathrm{calc}}$	$\lambda_{\exp}$
NiPor	Fluorescence	708	620–670 ^a^
NiCorr	Fluorescence	597	NA
PdPor	Fluorescence	475	550 ^b^, 564 ^c^, 560 ^e^
PdPor	Phosphorescence	703	640 ^b^, 663 ^c^, 658 ^d^, 664 ^e^, 657 ^g^
PdCorr	Fluorescence	412	515, 468 ^f^
PdCorr	Phosphorescence	657	702, 653 ^f^

^a^ Ref. [33],^b^ Ref. [42], ^c^ Ref. [9], ^d^ Ref. [43], ^e^ Ref. [50], ^f^ Ref. [15], ^g^ Ref. [10].

**Table 8:** SOCC values calculated with the TDDFT method for NiPor, NiCorr, PdPor and PdCorr (in cm^−1^) with the TDDFT/PBE0 and CPCM/methanol solvent model. For nickel complexes the def2-TZVP basis was used, while for palladium, ZORA-def2-TZVP was used.

NiPor			NiCorr			PdPor			PdCorr		
T1	S2	504	T1	S1	477	T5	S11	893	T2	S3	338
	S3	504		S3	492		S12	893		S5	142
T2	S1	515	T2	S2	512		S13	148	T3	S2	103
	S2	147		S3	293		S14	148		S3	268
	S3	260		S4	280	T6	S5	218		S7	146
	S4	312	T3	S1	292		S13	382	T4	S3	197
T3	S1	515		S2	489		S14	382	T5	S3	190
	S2	260		S4	314	T7	S10	186		S4	517
	S3	147	T4	S1	280		S11	166		S5	380
	S4	312		S3	312		S12	166		S9	105
T4	S2	309	T5	S7	202		S13	341	T6	S5	105
	S3	309	T7	S7	171		S14	341		S7	717
T10	S11	134	T8	S7	134		S5	452		S9	314
	S12	208		S6	251		S12	485	T7	S2	400
	S13	208					S14	137		S4	272
T12	S12	203					S15	874		S5	577
	S13	203				T9	S5	452		S7	168
T12	S12	203					S11	485		S8	148
	S13	203					S13	137		S9	242
T13	S9	127					S15	874	T8	S4	271
	S12	225				T10	S3	212		S5	428
	S13	225					S11	169		S7	663
T14	S10	141					S12	169		S9	459
	S12	225					S13	382	T9	S2	111
	S13	225					S14	382		S3	468
						T11	S4	248	T10	S1	138
							S11	205		S5	106
							S12	205		S7	200
							S13	373		S8	114
							S14	373		S9	158
									T11	S5	108
										S7	196
										S9	103
									T12	S2	103
										S3	330

**Table 9:** Electronic transitions of NiPor calculated with the NEVPT2 method (def2-TZVP basis set, CPCM/methanol solvent model).

No	E(eV)	λ(nm)	*f*	Orbitals	Weight
Singlet transitions					
1	1.86	663.3	0.000000	$d_{z^{2}}\to \pi_{4}^{*}$	0.68
2	1.87	662.9	0.000000	$d_{z^{2}}\to \pi_{3}^{*}$	0.68
3	2.0	619.6	0.000000	$d_{xz}\to \pi_{4}^{*}$	0.44
				$d_{yz}\to \pi_{3}^{*}$	0.2
4	2.02	611.6	0.000000	$d_{yz}\to d_{xy}$	0.87
5	2.02	611.6	0.000000	$\pi_{1}\to d_{xy}$	0.1
6	2.02	611.4	0.000000	$d_{xz}\to d_{xy}$	0.87
7	2.02	611.3	0.000000	$d_{xz}\to \pi_{3}^{*}$	0.40
				$d_{yz}\to \pi_{4}^{*}$	0.24
8	2.05	604.2	0.000000	$d_{z^{2}}\to d_{xy}$	0.88
9	2.07	597.8	0.000000	$d_{yz}\to \pi_{3}^{*}$	0.43
				$d_{xz}\to \pi_{4}^{*}$	0.19
10	2.12	583.2	0.000000	$d_{yz}\to \pi_{4}^{*}$	0.38
				$d_{xz}\to \pi_{3}^{*}$	0.23
11	2.39	517.1	0.047674	$\pi_{2}\to \pi_{3}^{*}$	0.30
				$\pi_{2}\to \pi_{4}^{*}$	0.26
				$\pi_{1}\to \pi_{4}^{*}$	0.19
				$\pi_{1}\to \pi_{3}^{*}$	0.16
12	2.39	517.1	0.047583	$\pi_{2}\to \pi_{4}^{*}$	0.30
				$\pi_{2}\to \pi_{3}^{*}$	0.26
				$\pi_{1}\to \pi_{3}^{*}$	0.19
				$\pi_{1}\to \pi_{4}^{*}$	0.16
13	2.48	498.6	0.000000	$d_{x^{2}-y^{2}}\to d_{xy}$	0.9
14	3.07	403.4	0.000065	$d_{x^{2}-y^{2}}\to \pi_{4}^{*}$	0.58
15	3.07	403.2	0.000071	$d_{x^{2}-y^{2}}\to \pi_{3}^{*}$	0.58
16	3.2	387.4	1.594977	$\pi_{1}\to \pi_{4}^{*}$	0.52
				$\pi_{2}\to \pi_{3}^{*}$	0.31
17	3.2	387.2	1.597797	$\pi_{1}\to \pi_{3}^{*}$	0.52
				$\pi_{2}\to \pi_{4}^{*}$	0.31
Triplet transitions					
1	0.231	5367.0	0.0000	$d_{z^{2}}\to d_{xy}$	0.9
2	0.295	4202.7	0.0000	$d_{yz}\to d_{xy}$	0.86
3	0.296	4188.5	0.0000	$d_{xz}\to d_{xy}$	0.86
4	1.176	1054.2	0.0000	$d_{x^{2}-y^{2}}\to d_{xy}$	0.83
5	2.058	602.42	0.0000	$\pi_{1}\to \pi_{4}^{*}$	0.86
6	2.059	602.13	0.0000	$\pi_{1}\to \pi_{3}^{*}$	0.86
7	2.315	535.55	0.0000	$\pi_{2}\to \pi_{4}^{*}$	0.86
8	2.317	535.08	0.0000	$\pi_{2}\to \pi_{3}^{*}$	0.86
9	2.614	474.29	0.0000	$d_{z^{2}}\to \pi_{4}^{*}$	0.43
10	2.687	461.40	0.0000	$d_{z^{2}}\to \pi_{3}^{*}$	0.44

**Table 10:** Electronic transitions of NiCorr calculated with the NEVPT2 method (def2-TZVP basis set, CPCM/methanol solvent model).

No	E(eV)	λ(nm)	*f*	Orbitals	Weight
Singlet transitions					
1	2.16	572.2	0.00013	$d_{z^{2}}\to d_{xy}$	0.67
				$d_{yz}+\pi_{1}\to d_{xy}$	0.23
2	2.19	565.9	0.00001	$d_{yz}+\pi_{1}\to d_{xy}$	0.56
				$d_{z^{2}}\to d_{xy}$	0.25
3	2.26	548.3	0.01332	$d_{z^{2}}\to \pi_{3}^{*}$	0.43
				$d_{yz}+\pi_{1}\to \pi_{3}^{*}$	0.23
4	2.28	541.9	0.00001	$d_{xz}\to d_{xy}$	0.83
5	2.44	507.9	0.00337	$d_{xz}\to \pi_{3}^{*}$	0.62
6	2.51	492.4	0.04073	$d_{yz}+\pi_{1}\to \pi_{3}^{*}$	0.43
				$d_{z^{2}}\to \pi_{3}^{*}$	0.25
7	2.68	462.3	0.31598	$\pi_{2}\to \pi_{3}^{*}$	0.75
8	3.51	352.5	0.02077	$d_{z^{2}}\to \pi_{4}^{*}$	0.33
				$d_{yz}+\pi_{1}\to \pi_{4}^{*}$	0.17
9	3.61	342.9	0.29217	$\pi_{2}\to \pi_{4}^{*}$	0.38
				$d_{xz}\to \pi_{4}^{*}$	0.19
10	3.62	342.4	0.02888	$d_{yz}+\pi_{1}\to \pi_{4}^{*}$	0.25
				$d_{z^{2}}\to \pi_{4}^{*}$	0.14
11	3.76	329.5	0.04108	$\pi_{1}+d_{yz}\to \pi_{3}^{*}$	0.49
12	4.06	305.4	0.04424	$d_{yz}+\pi_{1}\to \pi_{4}^{*}$	0.14
13	4.06	305.1	0.41735	$d_{xz}\to \pi_{4}^{*}$	0.26
				$\pi_{2}\to \pi_{4}^{*}$	0.17
Triplet transitions					
1	0.41	3023.9	0.000	$d_{z^{2}}\to d_{xz}$	0.93
2	0.52	2366.0	0.000	$d_{yz}+\pi_{1}\to d_{xz}$	0.72
3	0.58	2122.9	0.000	$d_{xz}\to d_{xz}$	0.82
4	2.47	501.94	0.000	$d_{z^{2}}\to \pi_{3}^{*}$	0.43
5	2.49	497.71	0.000	$d_{yz}+\pi_{1}\to \pi_{3}^{*}$	0.54
				$\pi_{1}+d_{yz}\to \pi_{3}^{*}$	0.18
6	2.55	484.67	0.000	$\pi_{2}\to \pi_{3}^{*}$	0.78
7	2.84	435.17	0.000	$d_{xz}\to \pi_{3}^{*}$	0.34
8	2.90	426.63	0.000	$d_{z^{2}}\to \pi_{3}^{*}$	0.25
9	2.99	414.09	0.000	$d_{xz}\to \pi_{3}^{*}$	0.23
10	3.05	406.35	0.000	$\pi_{2}\to \pi_{4}^{*}$	0.34
				$d_{yz}+\pi_{1}\to \pi_{3}^{*}$	0.14
11	3.30	375.01	0.000	$d_{yz}+\pi_{1}\to \pi_{4}^{*}$	0.46
12	3.47	356.98	0.000	$d_{z^{2}}\to \pi_{4}^{*}$	0.46
13	3.59	345.34	0.000	$d_{xz}\to \pi_{4}^{*}$	0.49
14	3.62	342.48	0.000	$d_{z^{2}}\to \pi_{4}^{*}$	0.22

**Table 11:** Electronic transitions of PdPor calculated with the NEVPT2 method (def2-TZVP basis set, CPCM/methanol solvent model).

No	E(eV)	λ(nm)	*f*	Orbitals	Weight
Singlet transitions					
1	2.45	507	0.0445	$\pi_{1}\to \pi_{3}^{*}$	0.4200
				$\pi_{2}\to \pi_{4}^{*}$	0.2720
				$\pi_{1}\to \pi_{4}^{*}$	0.1533
2	2.45	507	0.0445	$\pi_{1}\to \pi_{4}^{*}$	0.4200
				$\pi_{2}\to \pi_{3}^{*}$	0.2720
				$\pi_{1}\to \pi_{3}^{*}$	0.1533
3	3.11	399	0.0000	$d_{z^{2}}\to \pi_{3}^{*}$	0.8643
4	3.11	399	0.0000	$d_{z^{2}}\to \pi_{4}^{*}$	0.8643
5	3.22	385	0.0000	$d_{yz}\to \pi_{3}^{*}$	0.3596
				$d_{xz}\to \pi_{4}^{*}$	0.3580
6	3.22	385	0.0000	$d_{xz}\to \pi_{4}^{*}$	0.4107
				$d_{yz}\to \pi_{3}^{*}$	0.4091
7	3.26	380	1.6557	$\pi_{2}\to \pi_{3}^{*}$	0.3295
				$\pi_{2}\to \pi_{4}^{*}$	0.2089
				$\pi_{1}\to \pi_{4}^{*}$	0.2022
				$\pi_{1}\to \pi_{3}^{*}$	0.1282
8	3.26	380	1.6559	$\pi_{2}\to \pi_{4}^{*}$	0.3295
				$\pi_{2}\to \pi_{3}^{*}$	0.2089
				$\pi_{1}\to \pi_{3}^{*}$	0.2022
				$\pi_{1}\to \pi_{4}^{*}$	0.1282
9	3.27	380	0.0000	$d_{yz}\to \pi_{4}^{*}$	0.3555
				$d_{xz}\to \pi_{3}^{*}$	0.3553
10	3.50	354	0.0000	$d_{xz}\to \pi_{3}^{*}$	0.3935
				$d_{yz}\to \pi_{4}^{*}$	0.3934
11	4.10	303	0.0000	$d_{z^{2}}\to d_{xy}$	0.9043
12	4.11	302	0.0000	$\pi_{2}\to d_{xy}$	0.4127
13	4.18	296	0.0000	$\pi_{1}\to d_{xy}$	0.3843
14	4.24	292	0.0000	$d_{x^{2}-y^{2}}\to \pi_{3}^{*}$	0.8247
15	4.24	292	0.0000	$d_{x^{2}-y^{2}}\to \pi_{4}^{*}$	0.8247
16	4.25	292	0.0000	$d_{xz}\to d_{xy}$	0.8972
Triplet transitions					
1	2.14	579.3	0.0000	$\pi_{2}\to \pi_{4}^{*}$	0.685
				$\pi_{2}\to \pi_{3}^{*}$	0.203
2	2.14	579.3	0.0000	$\pi_{2}\to \pi_{3}^{*}$	0.685
				$\pi_{2}\to \pi_{4}^{*}$	0.203
3	2.34	529.1	0.0000	$\pi_{1}\to \pi_{4}^{*}$	0.882
4	2.34	529.1	0.0000	$\pi_{1}\to \pi_{3}^{*}$	0.882
5	3.07	403.8	0.0000	$d_{z^{2}}\to \pi_{3}^{*}$	0.866
6	3.07	403.8	0.0000	$d_{z^{2}}\to \pi_{4}^{*}$	0.866
7	3.14	394.7	0.0000	$d_{yz}\to \pi_{4}^{*}$	0.426
				$d_{xz}\to \pi_{3}^{*}$	0.424
8	3.15	393.2	0.0000	$d_{xz}\to \pi_{4}^{*}$	0.399
				$d_{yz}\to \pi_{3}^{*}$	0.396
9	3.16	392.8	0.0000	$d_{z^{2}}\to d_{xy}$	0.913
10	3.17	391.1	0.0000	$d_{xz}\to \pi_{3}^{*}$	0.398
				$d_{yz}\to \pi_{4}^{*}$	0.397
11	3.17	390.7	0.0000	$d_{yz}\to \pi_{3}^{*}$	0.420
				$d_{xz}\to \pi_{4}^{*}$	0.418
13	3.31	374.6	0.0000	$d_{yz}\to d_{xy}$	0.869
14	3.31	374.6	0.0000	$d_{xz}\to d_{xy}$	0.869
15	4.01	309.3	0.0000	$d_{x^{2}-y^{2}}\to d_{xy}$	0.898
16	4.09	302.8	0.0000	$\pi_{2}\to d_{xy}$	0.415

**Table 12:** Electronic transitions of PdCorr calculated with the NEVPT2 method (def2-TZVP basis set, CPCM/methanol solvent model).

No	E(eV)	λ(nm)	*f*	Orbitals	Weight
Singlet transitions					
1	3.244	382.1	0.1779	$d_{z^{2}}\to \pi_{3}^{*}$	0.78
2	3.369	368.0	0.1155	$\pi_{2}\to \pi_{3}^{*}$	0.58
				$d_{z^{2}}\to d_{xy}$	0.20
				$\pi_{1}+d_{yz}\to \pi_{4}^{*}$	0.11
3	3.474	356.8	0.0024	$d_{z^{2}}\to \pi_{3}^{*}$	0.89
4	3.843	322.6	0.0788	$d_{xz}\to \pi_{3}^{*}$	0.87
5	4.145	299.1	0.1609	$\pi_{1}+d_{yz}\to \pi_{4}^{*}$	0.54
				$d_{z^{2}}\to \pi_{4}^{*}$	0.18
6	4.446	278.8	0.0574	$d_{z^{2}}\to \pi_{4}^{*}$	0.71
				$\pi_{1}+d_{yz}\to \pi_{4}^{*}$	0.14
7	4.512	274.7	0.1042	$d_{z^{2}}\to d_{xy}$	0.70
				$\pi_{2}\to \pi_{3}^{*}$	0.21
8	4.533	273.5	0.6477	$d_{yz}+\pi_{1}\to \pi_{3}^{*}$	0.54
				$\pi_{2}\to \pi_{3}^{*}$	0.29
9	4.769	259.9	0.0042	$\pi_{1}+d_{yz}\to d_{xy}$	0.56
				$d_{yz}+\pi_{1}\to d_{xy}$	0.32
10	4.807	257.9	0.0531	$\pi_{2}\to d_{xy}$	0.56
11	5.470	226.6	0.0394	$d_{yz}+\pi_{1}\to \pi_{4}^{*}$	0.71
Triplet transitions					
1	2.822	439.3	0.0000	$\pi_{1}+d_{yz}\to \pi_{3}^{*}$	0.67
				$\pi_{2}\to \pi_{4}^{*}$	0.24
2	2.868	432.2	0.0000	$\pi_{2}\to \pi_{3}^{*}$	0.70
				$\pi_{1}+d_{yz}\to d_{xy}$	0.20
3	3.470	357.2	0.0000	$d_{z^{2}}\to \pi_{3}^{*}$	0.90
4	3.579	346.4	0.0000	$\pi_{2}\to \pi_{4}^{8}$	0.37
				$d_{yz}+\pi_{1}\to \pi_{3}^{*}$	0.25
				$\pi +d_{yz}\to \pi_{3}^{*}$	0.22
5	3.645	340.1	0.0000	$d_{xz}\to \pi_{3}^{*}$	0.73
				$\pi_{1}+d_{yz}\to \pi_{4}$	0.11
6	3.919	316.3	0.0000	$\pi_{1}+d_{yz}\to \pi_{4}^{8}$	0.45
				$d_{z^{2}}\to \pi_{3}^{*}$	0.20
				$\pi_{2}\to \pi_{3}^{*}$	0.12
7	4.098	302.5	0.0000	$d_{z^{2}}\to d_{xy}$	0.93
8	4.124	300.6	0.0000	$\pi_{1}+d_{yz}\to d_{xy}$	0.47
				$d_{yz}+\pi_{1}\to d_{xy}$	0.44
9	4.389	282.4	0.0000	$d_{xz}\to d_{xy}$	0.92
10	4.415	280.8	0.0000	$d_{yz}+\pi_{1}\to \pi_{3}^{*}$	0.54
				$\pi_{2}\to \pi_{4}\to d_{xy}$	0.20
11	4.469	277.4	0.0000	$d_{z^{2}}\to \pi_{4}^{*}$	0.89
12	4.666	265.7	0.0000	$d_{xz}\to \pi_{4}^{*}$	0.80
13	4.742	261.4	0.0000	$\pi_{2}\to d_{xy}$	0.56
14	5.588	221.8	0.0000	$d_{yz}+\pi_{1}\to d_{xy}$	0.42
15	5.957	208.1	0.0000	$d_{yz}+\pi_{1}\to d_{xy}$	0.25
				$\pi_{1}+d_{yz}\to d_{xy}$	0.16
16	6.014	206.1	0.0000	$d_{yz}+\pi_{1}\to \pi_{4}^{*}$	0.19

**Table 13:** HOMO–LUMO and $d_{x^{2}-y^{2}}-d_{z^{2}}$ energy differences for nickel and palladium porphyrin and corrin.

Molecule	ΔE(H-L) (eV)	$\Delta E(d_{x^{2}-y^{2}}-d_{z^{2}})$ (eV)
NiPor	3.38	5.82
NiCorr	4.16	6.34
PdPor	3.43	6.77
PdCorr	4.11	7.11

**Table 14:** Racah parameters for Ni(II) and Pd(II) ions calculated with the AILFT method.

Molecule	B (eV)	C (eV)	4B+C (eV)
Ni(II)	0.165	0.603	1.263
Pd(II)	0.119	0.462	0.938

## Data Availability

The original data presented in the study are openly available in [RepOD] at [https://repod.icm.edu.pl/dataset.xhtml?token=867ecd1f-e6d3-4843-9333-687a8038c974[] accessed on 29 January 2026.

## Supplementary Materials

The following supporting information can be downloaded at: https://www.mdpi.com/article/10.3390/ijms27031577/s1.

## Abbreviations

The following abbreviations are used in this manuscript:

DFT	Density Functional Theory
TDDFT	Time Dependent Density Functional Theory
NEVPT2	N-Electron Valence State Perturbation Theory
NiPor	Nickel(II) Porphyrrin
NiCorr	Nickel(II) Corrin
PdPor	Palladium(II) Porphyrin
PdCorr	Palladium(II) Corrin)
SOC	Spin–Orbit Coupling
SOCC	Spin–Orbit Coupling Coefficient
IC	Internal Conversion
ISC	Intersystem Crossing
$S_{Q}$	Q Band
$S_{Soret}$	Soret Band
MECP	Minimum Energy Crossing Point
