# Supplementary material for: Photophysical Processes of Porphyrin and Corrin Complexes with Nickel and Palladium
Source: Int J Mol Sci. 2026 Feb 5;27(3):1577. doi: 10.3390/ijms27031577 (PMC12898465; doi:10.3390/ijms27031577)
Supplement: Supplementary file 1 [file ijms-27-01577-s001.zip › ijms-4089915-supplementary.pdf]

PHOTOPHYSICAL PROCESSES OF PORPHYRIN AND CORRIN COMPLEXES WITH NICKEL AND PALLADIUM

Maria Jaworska. Piotr Lodowski

Institute of Chemistry. University of Silesia in Katowice. Szkolna 9. PL 40-006 Katowice. Poland

Supplementary Materials

Supplementary Tables.

Table S1. The twenty lowest. vertical singlet electronic transitions for the PdPor complex based on the TD-DFT/PBE0/ZORA-def2-TZVP.SARC-ZORA-TZVP calculations with CPCM/Methanol solvent model. The geometry was fully optimised in ZORA-def2-TZVP/SARC-ZORA-TZVP basis set.

	E(eV)	λ(nm)	*f*	%	MOs		Character
S_1	2.62	473	0.0079	46	103 → 104	H → L	$\pi_1 \rightarrow \pi_y$*
				41	102 → 105	H-1 → L+1	$\pi_2 \rightarrow \pi_x$*
S_2	2.62	473	0.0079	46	103 → 105	H → L+1	$\pi_1 \rightarrow \pi_x$*
				41	102 → 104	H-1 → L	$\pi_2 \rightarrow \pi_y$*
S_3	3.40	365	0.0000	40	100 → 104	H-3 → L	$\pi/d_{xz} \rightarrow \pi_y$*
				40	101 → 105	H-2 → L+1	$\pi/d_{yz} \rightarrow \pi_x$*
S_4	3.45	359	0.0000	47	100 → 104	H-3 → L	$\pi/d_{xz} \rightarrow \pi_y$*
				47	101 → 105	H-2 → L+1	$\pi/d_{yz} \rightarrow \pi_x$*
S_5	3.49	355	0.0000	38	100 → 105	H-3 → L+1	$\pi/d_{xz} \rightarrow \pi_x$*
				38	101 → 104	H-2 → L	$\pi/d_{yz} \rightarrow \pi_y$*
				17	99 → 106	H-4 → L+2	$d_{z2} \rightarrow d_{xy}$-n
S_6	3.54	350	0.0000	99	102 → 106	H-1 → L+2	$\pi_2 \rightarrow d_{xy}$-n
S_7	3.60	345	0.0000	100	103 → 106	H → L+2	$\pi_1 \rightarrow d_{xy}$-n
S_8	3.76	330	1.6663	27	102 → 105	H-1 → L+1	$\pi_2 \rightarrow \pi_x$*
				25	103 → 104	H → L	$\pi_1 \rightarrow \pi_y$*
				17	102 → 104	H-1 → L	$\pi_2 \rightarrow \pi_y$*
				16	103 → 105	H → L+1	$\pi_1 \rightarrow \pi_x$*
S_9	3.76	330	1.6663	27	102 → 104	H-1 → L	$\pi_2 \rightarrow \pi_y$*
				25	103 → 105	H → L+1	$\pi_1 \rightarrow \pi_x$*
				17	102 → 105	H-1 → L+1	$\pi_2 \rightarrow \pi_x$*
				16	103 → 104	H → L	$\pi_1 \rightarrow \pi_y$*
S_{10}	3.79	327	0.0000	40	100 → 105	H-3 → L+1	$\pi/d_{xz} \rightarrow \pi_x$*
				40	101 → 104	H-2 → L	$\pi/d_{yz} \rightarrow \pi_y$*
S_{11}	3.81	326	0.0002	79	101 → 106	H-2 → L+2	$\pi/d_{yz} \rightarrow d_{xy}$-n
				11	92 → 106	H-11 → L+2	$d_{yz}/\pi \rightarrow d_{xy}$-n
S_{12}	3.81	326	0.0002	79	100 → 106	H-3 → L+2	$\pi/d_{xz} \rightarrow d_{xy}$-n
				11	93 → 106	H-10 → L+2	$d_{xz}/\pi \rightarrow d_{xy}$-n
S_{13}	3.84	323	0.0001	89	99 → 104	H-4 → L	$d_{z2} \rightarrow \pi_y$*
S_{14}	3.84	323	0.0001	89	99 → 105	H-4 → L+1	$d_{z2} \rightarrow \pi_x$*
S_{15}	3.92	317	0.0000	80	99 → 106	H-4 → L+2	$d_{z2} \rightarrow d_{xy}$-n
S_{16}	4.20	295	0.0611	91	98 → 104	H-5 → L	$\pi \rightarrow \pi_y$*
S_{17}	4.20	295	0.0611	91	98 → 105	H-5 → L+1	$\pi \rightarrow \pi_x$*
S_{18}	4.54	273	0.0000	90	102 → 107	H-1 → L+3	$\pi_2 \rightarrow \pi$*
S_{19}	4.55	272	0.0000	90	103 → 107	H → L+3	$\pi_1 \rightarrow \pi$*
S_{20}	4.57	271	0.2471	88	97 → 104	H-6 → L	$\pi \rightarrow \pi_y$*

Table S2. Calculated values of spin-orbit coupling constant (SOCC) for selected states of NiCorr complex.

States		SOC [cm^{-1}]						SOCC [cm^{-1}]
Triplet	Singlet	Z		X		Y		
		$\Re$e	$\Im$m	$\Re$e	$\Im$m	$\Re$e	$\Im$m	
T_1	S_1	0	0	0	477	0	0	477
	S_2	0	0	0	-30	0	0	30
	S_3	0	53	0	0	0	489	492
	S_4	0	0	0	-85	0	0	85
T_2	S_2	0	0	0	512	0	0	512
	S_3	0	287	0	0	0	-57	293
	S_4	0	0	0	-280	0	0	280
	S_5	0	0	0	-24	0	0	24
	S_6	0	0	0	0	0	37	37
T_3	S_1	0	-270	0	0	0	109	292
	S_2	0	69	0	0	0	484	489
	S_4	0	68	0	0	0	307	314
	S_5	0	9	0	0	0	78	78
T_4	S_1	0	0	0	280	0	0	280
	S_2	0	0	0	-56	0	0	56
	S_3	0	-41	0	0	0	-310	312
	S_4	0	0	0	-28	0	0	28
T_5	S_1	0	-10	0	0	0	32	34
	S_7	0	0	0	202	0	0	202
T_7	S_1	0	0	0	-32	0	0	32
	S_3	0	1	0	0	0	-35	35
	S_6	0	-63	0	0	0	20	66
	S_7	0	-18	0	0	0	-170	171
T_8	S_6	0	0	0	23	0	0	23
	S_7	0	0	0	-134	0	0	134
	S_2	0	7	0	0	0	-94	94
	S_6	0	0	0	-251	0	0	251

Table S3. Calculated values of spin-orbit coupling constant (SOCC) for selected states of NiPor complex.

States		SOC [cm^{-1}]						SOCC [cm^{-1}]
Triplet	Singlet	Z		X		Y		
		$\Re$e	$\Im$m	$\Re$e	$\Im$m	$\Re$e	$\Im$m	
T_1	S_2	0	0	0	-478	0	-159	504
	S_3	0	0	0	-159	0	478	504
	S_{12}	0	0	0	20	0	23	31
	S_{13}	0	0	0	-23	0	20	31
T_2	S_1	0	0	0	506	0	-99	515
	S_2	0	-147	0	0	0	0	147
	S_3	0	260	0	0	0	0	260
	S_4	0	0	0	-305	0	-63	312
	S_9	0	0	0	-26	0	-5	27
	S_{10}	0	0	0	-4	0	-22	22
	S_{11}	0	0	0	-29	0	6	29
T_3	S_1	0	0	0	-99	0	-506	515
	S_2	0	-260	0	0	0	0	260
	S_3	0	-147	0	0	0	0	147
	S_4	0	0	0	63	0	-305	312
	S_9	0	0	0	5	0	-26	27
	S_{10}	0	0	0	-22	0	4	22
	S_{11}	0	0	0	6	0	29	29
T_4	S_2	0	0	0	294	0	-95	309
	S_3	0	0	0	95	0	294	309
T_5	S_7	0	0	0	-16	0	14	21
	S_8	0	0	0	15	0	18	23
T_6	S_7	0	0	0	-14	0	-16	21
	S_8	0	0	0	-18	0	15	23
T_7	S_7	0	0	0	-13	0	19	23
	S_8	0	0	0	-18	0	-13	22
T_8	S_7	0	0	0	-19	0	-13	23
	S_8	0	0	0	13	0	-18	22
T_9	S_5	0	0	0	-17	0	-15	22
	S_6	0	0	0	-15	0	17	22
	S_{14}	0	0	0	17	0	20	26
	S_{15}	0	0	0	-20	0	17	26
T_{10}	S_{11}	0	134	0	0	0	0	134
	S_{12}	0	0	0	-159	0	134	208
	S_{13}	0	0	0	-134	0	-159	208
T_{11}	S_5	0	0	0	-16	0	18	24
	S_6	0	0	0	18	0	16	24
	S_{14}	0	0	0	-15	0	13	20
	S_{15}	0	0	0	-13	0	-15	20
T_{12}	S_2	0	0	0	28	0	-9	30
	S_3	0	0	0	9	0	28	30
	S_{12}	0	0	0	-132	0	154	203
	S_{13}	0	0	0	154	0	132	203
T_{13}	S_2	0	0	0	-7	0	-21	22
	S_3	0	0	0	21	0	-7	22
	S_9	0	-127	0	0	0	0	127
	S_{12}	0	0	0	171	0	147	225
	S_{13}	0	0	0	147	0	-171	225
T_{14}	S_2	0	0	0	29	0	10	31
	S_3	0	0	0	10	0	-29	31
	S_{10}	0	141	0	0	0	0	141
	S_{12}	0	0	0	-145	0	-172	225
	S_{13}	0	0	0	172	0	-145	225

Table S4. Calculated values of spin-orbit coupling constant (SOCC) for selected states of PdCorr complex.

States		SOC [cm^{-1}]						SOCC [cm^{-1}]
Triplet	Singlet	Z		X		Y		
		$\Re$e	$\Im$m	$\Re$e	$\Im$m	$\Re$e	$\Im$m	
T_1	S_5	0	0	0	-20	0	0	20
	S_6	0	-30	0	0	0	94	99
	S_7	0	0	0	-20	0	0	20
	S_8	0	-12	0	0	0	-34	36
T_2	S_3	0	0	0	338	0	0	338
	S_4	0	-33	0	0	0	-64	72
	S_5	0	-122	0	0	0	72	142
	S_6	0	0	0	-34	0	0	34
	S_7	0	19	0	0	0	35	40
	S_9	0	66	0	0	0	-19	69
T_3	S_2	0	96	0	0	0	-36	103
	S_3	0	23	0	0	0	267	268
	S_4	0	0	0	-29	0	0	29
	S_6	0	-33	0	0	0	34	47
	S_7	0	0	0	146	0	0	146
	S_8	0	-47	0	0	0	2	47
	S_9	0	0	0	-80	0	0	80
T_4	S_1	0	-27	0	0	0	22	35
	S_3	0	0	0	-197	0	0	197
	S_4	0	40	0	1	0	23	46
	S_5	0	72	0	0	0	-14	73
	S_6	0	0	0	-83	0	0	83
	S_7	0	-10	0	0	0	-35	36
	S_8	0	0	0	67	0	0	67
	S_9	0	-41	0	0	0	-1	41
T_5	S_1	0	0	0	-24	0	0	24
	S_3	0	-20	0	0	0	189	190
	S_4	0	0	0	-517	0	0	517
	S_5	0	0	0	380	0	0	380
	S_6	0	16	0	-1	0	93	95
	S_7	0	0	0	54	0	0	54
	S_8	0	0	0	0	0	48	48
	S_9	0	0	0	105	0	0	105
T_6	S_1	0	0	0	-42	0	0	42
	S_2	0	-7	0	0	0	95	96
	S_3	0	23	0	0	0	50	55
	S_4	0	0	0	72	0	0	72
	S_5	0	0	0	105	0	0	105
	S_6	0	48	0	1	0	-54	73
	S_7	0	0	0	-717	0	0	717
	S_8	0	17	0	0	0	-29	34
	S_9	0	0	0	314	0	0	314
T_7	S_1	0	-4	0	0	0	12	13
	S_2	0	0	0	-400	0	0	400
	S_4	0	-27	0	0	0	-271	272
	S_5	0	-44	0	0	0	-575	577
	S_6	0	0	0	72	0	1	72
	S_7	0	-18	0	0	0	167	168
	S_8	0	0	0	148	0	0	148
	S_9	0	49	0	0	0	237	242
T_8	S_3	0	0	0	45	0	1	45
	S_4	0	259	0	0	0	-80	271
	S_5	0	-194	0	0	0	382	428
	S_7	0	-149	0	0	0	-646	663
	S_8	0	0	0	54	0	0	54
	S_9	0	47	0	0	0	456	459
T_9	S_1	0	0	0	19	0	0	19
	S_2	0	105	0	0	0	-36	111
	S_3	0	41	0	0	0	466	468
	S_4	0	0	0	76	0	0	76

	S_5	0	0	0	-73	0	0	73
	S_6	0	-24	0	0	0	-10	26
	S_7	0	0	0	62	0	0	62
	S_8	0	-30	0	0	0	18	35
	S_9	0	0	0	-23	0	0	23
T_{10}	S_1	0	21	0	0	0	-136	138
	S_2	0	0	0	-62	0	0	62
	S_3	0	0	0	-65	0	-1	65
	S_4	0	93	0	0	0	-8	93
	S_5	0	-40	0	0	0	99	106
	S_6	0	0	0	-71	0	0	71
	S_7	0	-57	0	0	0	-192	200
	S_8	0	0	0	-114	0	0	114
	S_9	0	-3	0	0	0	158	158
T_{11}	S_3	0	0	0	72	0	0	72
	S_4	0	-54	0	0	0	-22	59
	S_5	0	22	0	0	0	-106	108
	S_6	0	0	0	-40	0	0	40
	S_7	0	120	0	0	0	154	196
	S_9	0	90	0	0	0	-51	103
T_{12}	S_2	0	102	0	0	0	-12	103
	S_3	0	31	0	0	0	329	330
	S_4	0	0	0	-39	0	0	39
	S_5	0	0	0	37	0	0	37
	S_6	0	-27	0	0	0	-4	27
	S_8	0	-34	0	0	0	22	40

Table S5. Calculated values of spin-orbit coupling constant (SOCC) for selected states of PdPor complex.

States		SOC [cm⁻¹]						SOCC [cm⁻¹]
Triplet	Singlet	Z		X		Y		
		$\Re$e	$\Im$m	$\Re$e	$\Im$m	$\Re$e	$\Im$m	
T_1	S_6	0	0	0	9	0	72	72
	S_7	0	0	0	-45	0	6	45
T_2	S_6	0	0	0	-72	0	9	72
	S_7	0	0	0	-6	0	-45	45
T_3	S_6	0	0	0	40	0	-18	44
	S_7	0	0	0	-32	0	-70	77
T_4	S_6	0	0	0	-18	0	-40	44
	S_7	0	0	0	-70	0	32	77
T_5	S_{11}	0	0	0	618	0	644	893
	S_{12}	0	0	0	-644	0	618	893
	S_{13}	0	0	0	-84	0	-123	148
	S_{14}	0	0	0	123	0	-84	148
T_6	S_5	0	-218	0	0	0	0	218
	S_{11}	0	0	0	-33	0	31	46
	S_{12}	0	0	0	-32	0	-33	46
	S_{13}	0	0	0	-316	0	216	382
	S_{14}	0	0	0	-216	0	-316	382
	S_{15}	0	-93	0	0	0	0	93
T_7	S_{10}	0	-186	0	0	0	0	186
	S_{11}	0	0	0	-116	0	119	166
	S_{12}	0	0	0	119	0	116	166
	S_{13}	0	0	0	-195	0	280	341
	S_{14}	0	0	0	280	0	195	341
T_8	S_3	0	0	0	-36	0	41	54
	S_4	0	0	0	46	0	-40	61
	S_5	0	0	0	295	0	343	452
	S_{10}	0	0	0	56	0	49	74
	S_{11}	0	26	0	0	0	0	26
	S_{12}	0	-485	0	0	0	0	485
	S_{14}	0	137	0	0	0	0	137
	S_{15}	0	0	0	-570	0	-662	874
T_9	S_3	0	0	0	41	0	36	54
	S_4	0	0	0	40	0	46	61
	S_5	0	0	0	-343	0	295	452
	S_{10}	0	0	0	49	0	-56	74
	S_{11}	0	485	0	0	0	0	485
	S_{12}	0	26	0	0	0	0	26
	S_{13}	0	-137	0	0	0	0	137
	S_{15}	0	0	0	662	0	-570	874
T_{10}	S_3	0	212	0	0	0	0	212
	S_{11}	0	0	0	121	0	118	169
	S_{12}	0	0	0	118	0	-121	169
	S_{13}	0	0	0	313	0	219	382
	S_{14}	0	0	0	218	0	-313	382
T_{11}	S_4	0	-248	0	0	0	0	248
	S_{11}	0	0	0	-142	0	-148	205
	S_{12}	0	0	0	148	0	-142	205
	S_{13}	0	0	0	-210	0	-308	373
	S_{14}	0	0	0	308	0	-210	373
T_{12}	S_1	0	0	0	62	0	-10	63
	S_2	0	0	0	10	0	62	63
	S_8	0	0	0	74	0	27	79
	S_9	0	0	0	-27	0	74	79
T_{15}	S_1	0	0	0	-11	0	-64	65
	S_2	0	0	0	64	0	-11	65
	S_8	0	0	0	-19	0	52	55
	S_9	0	0	0	-52	0	-19	55
T_{16}	S_5	0	27	0	0	0	0	27
	S_{13}	0	0	0	40	0	-27	48
	S_{14}	0	0	0	27	0	40	48

Table S6. Calculated values of spin-orbit coupling constant (SOCC) for selected states of nickel and palladium complexes with porphyrin and corrin.

NiPor			NiCorr			PdPor			PdCorr		
States		SOCC [cm-1]	States		SOCC [cm-1]	States		SOCC [cm-1]	States		SOCC [cm-1]
Triplet	Singlet		Triplet	Singlet		Triplet	Singlet		Triplet	Singlet	
T_1	S_2	504	T_1	S_1	477	T_1	S_6	72	T_1	S_5	20
	S_3	504		S_2	30		S_7	45		S_6	99
	S_{12}	31		S_3	492	T_2	S_6	72		S_7	20
	S_{13}	31		S_4	85		S_7	45		S_8	36
T_2	S_1	515	T_2	S_2	512	T_3	S_6	44	T_2	S_3	338
	S_2	147		S_3	293		S_7	77		S_4	72
	S_3	260		S_4	280	T_4	S_6	44		S_5	142
	S_4	312		S_5	24		S_7	77		S_6	34
	S_9	27		S_6	37	T_5	S_{11}	893		S_7	40
	S_{10}	22	T_3	S_1	292		S_{12}	893		S_9	69
	S_{11}	29		S_2	489		S_{13}	148	T_3	S_2	103
T_3	S_1	515		S_4	314		S_{14}	148		S_3	268
	S_2	260		S_5	78	T_6	S_5	218		S_4	29
	S_3	147	T_4	S_1	280		S_{11}	46		S_6	47
	S_4	312		S_2	56		S_{12}	46		S_7	146
	S_9	27		S_3	312		S_{13}	382		S_8	47
	S_{10}	22		S_4	28		S_{14}	382		S_9	80
	S_{11}	29	T_5	S_1	34		S_{15}	93	T_4	S_1	35
T_4	S_2	309		S_7	202	T_7	S_{10}	186		S_3	197
	S_3	309	T_7	S_1	32		S_{11}	166		S_4	46
T_5	S_7	21		S_3	35		S_{12}	166		S_5	73
	S_8	23		S_6	66		S_{13}	341		S_6	83
T_6	S_7	21		S_7	171		S_{14}	341		S_7	36
	S_8	23	T_8	S_6	23	T_8	S_3	54		S_8	67
T_7	S_7	23		S_7	134		S_4	61		S_9	41
	S_8	22		S_2	94		S_5	452	T_5	S_1	24
T_8	S_7	23		S_6	251		S_{10}	74		S_3	190
	S_8	22					S_{11}	26		S_4	517
T_9	S_5	22					S_{12}	485		S_5	380
	S_6	22					S_{14}	137		S_6	95
	S_{14}	26					S_{15}	874		S_7	54
	S_{15}	26				T_9	S_3	54		S_8	48
T_{10}	S_{11}	134					S_4	61		S_9	105
	S_{12}	208					S_5	452	T_6	S_1	42
	S_{13}	208					S_{10}	74		S_2	96
T_{11}	S_5	24					S_{11}	485		S_3	55
	S_6	24					S_{12}	26		S_4	72
	S_{14}	20					S_{13}	137		S_5	105
	S_{15}	20					S_{15}	874		S_6	73
T_{12}	S_2	30				T_{10}	S_3	212		S_7	717
	S_3	30					S_{11}	169		S_8	34
	S_{12}	203					S_{12}	169		S_9	314
	S_{13}	203					S_{13}	382	T_7	S_1	13
T_{13}	S_2	22					S_{14}	382		S_2	400
	S_3	22				T_{11}	S_4	248		S_4	272
	S_9	127					S_{11}	205		S_5	577
	S_{12}	225					S_{12}	205		S_6	72
	S_{13}	225					S_{13}	373		S_7	168
T_{14}	S_2	31					S_{14}	373		S_8	148
	S_3	31				T_{12}	S_1	63		S_9	242
	S_{10}	141					S_2	63	T_8	S_3	45
	S_{12}	225					S_8	79		S_4	271
	S_{13}	225					S_9	79		S_5	428
						T_{15}	S_1	65		S_7	663
							S_2	65		S_8	54
							S_8	55		S_9	459
							S_9	55	T_9	S_1	19
						T_{16}	S_5	27		S_2	111
							S_{13}	48		S_3	468
							S_{14}	48		S_4	76

	S_5	73
	S_6	26
	S_7	62
	S_8	35
	S_9	23
T_{10}	S_1	138
	S_2	62
	S_3	65
	S_4	93
	S_5	106
	S_6	71
	S_7	200
	S_8	114
	S_9	158
T_{11}	S_3	72
	S_4	59
	S_5	108
	S_6	40
	S_7	196
	S_9	103
T_{12}	S_2	103
	S_3	330
	S_4	39
	S_5	37
	S_6	27
	S_8	40

Table S7. CASSCF (NEVPT2 diagonal energies) UV. ABSORPTION SPECTRUM VIA TRANSITION ELECTRIC DIPOLE MOMENTS

NEVPT2 State	GS		CASSCF state	Excitation Energy (eV) (NEVPT2)	λ(nm) (NEVPT2)	*f* (NEVPT2)
1	0-1A	->	29-1A	1.869132	663.3	0.000000371
2	0-1A	->	28-1A	1.870382	662.9	0.000000579
3	0-1A	->	37-1A	2.001191	619.6	0.000000006
4	0-1A	->	2-1A	2.027273	611.6	0.000000000
5	0-1A	->	13-1A	2.027276	611.6	0.000000000
6	0-1A	->	3-1A	2.027823	611.4	0.000000000
7	0-1A	->	36-1A	2.028227	611.3	0.000000005
8	0-1A	->	4-1A	2.052126	604.2	0.000000000
9	0-1A	->	35-1A	2.074048	597.8	0.000000010
10	0-1A	->	38-1A	2.125792	583.2	0.000000011
11	0-1A	->	5-1A	2.163365	573.1	0.000000000
12	0-1A	->	17-1A	2.340960	529.6	0.000000360
13	0-1A	->	16-1A	2.342896	529.2	0.000000403
14	0-1A	->	14-1A	2.397762	517.1	0.047674999
15	0-1A	->	15-1A	2.398132	517.0	0.047583555
16	0-1A	->	18-1A	2.408402	514.8	0.000000000
17	0-1A	->	19-1A	2.408870	514.7	0.000000001
18	0-1A	->	20-1A	2.426870	510.9	0.000000000
19	0-1A	->	1-1A	2.486431	498.6	0.000000000
20	0-1A	->	7-1A	2.539823	488.2	0.000000140
21	0-1A	->	6-1A	2.541491	487.8	0.000000140
22	0-1A	->	8-1A	2.568675	482.7	0.000000000
23	0-1A	->	9-1A	2.633238	470.8	0.000000000
24	0-1A	->	10-1A	2.660507	466.0	0.000000000
25	0-1A	->	47-1A	3.073234	403.4	0.000065146
26	0-1A	->	46-1A	3.074810	403.2	0.000071341
27	0-1A	->	41-1A	3.200476	387.4	1.594977304
28	0-1A	->	42-1A	3.201914	387.2	1.597797507
29	0-1A	->	23-1A	3.257142	380.7	0.000002982
30	0-1A	->	22-1A	3.257209	380.6	0.000002943
31	0-1A	->	12-1A	3.482868	356.0	0.000001848
32	0-1A	->	11-1A	3.483757	355.9	0.000001690
33	0-1A	->	39-1A	4.200901	295.1	0.000000151
34	0-1A	->	24-1A	4.259711	291.1	0.000000000
35	0-1A	->	25-1A	4.361679	284.3	0.000000000
6	0-1A	->	48-1A	4.484216	276.5	0.000000741
37	0-1A	->	49-1A	4.542855	272.9	0.000000020
38	0-1A	->	21-1A	4.614159	268.7	0.000000000
39	0-1A	->	26-1A	4.669542	265.5	0.000000061
40	0-1A	->	27-1A	4.669936	265.5	0.000000021
41	0-1A	->	40-1A	4.697494	263.9	0.000000353
42	0-1A	->	30-1A	4.706217	263.4	0.000000001

43	0-1A	->	31-1A	4.719910	262.7	0.000000002
44	0-1A	->	34-1A	4.763554	260.3	0.000000025
45	0-1A	->	33-1A	4.830526	256.7	0.000000389
46	0-1A	->	32-1A	4.831970	256.6	0.000000373
47	0-1A	->	43-1A	4.875719	254.3	0.010641715
48	0-1A	->	44-1A	4.876030	254.3	0.010090322
49	0-1A	->	45-1A	5.074840	244.3	0.000000003

Table S8. The lowest, vertical singlet and triplet electronic transitions for PdPor complex based on the SA-CASSCF(14,13)/NEVPT2/def2-TZVP calculations with CPCM/methanol solvent model.

	E(eV)	λ(nm)	*f*	% CFS	CSF													Character
					n	$3d_{x2-y2}$	$3d_{xz}$	$3d_{yz}$	$3d_{z2}$	π_2	π_1	$3d_{xy}$-n	π_x*	π_y*	$4d_{xz}$	$4d_{yz}$	$4d_{z2}$	
S_1	2.45	506	0.0463	51	2	2	2	2	2	2	1	0	0	1	0	0	0	$\pi_1 \rightarrow \pi_y$*
				33	2	2	2	2	2	1	2	0	1	0	0	0	0	$\pi_2 \rightarrow \pi_x$*
S_2	2.45	506	0.0463	51	2	2	2	2	2	2	1	0	1	0	0	0	0	$\pi_1 \rightarrow \pi_x$*
				33	2	2	2	2	2	1	2	0	0	1	0	0	0	$\pi_2 \rightarrow \pi_y$*
S_3	3.08	403	0.0000	87	2	2	2	2	1	2	2	0	0	1	0	0	0	$d_{z2} \rightarrow \pi_y$*
S_4	3.08	403	0.0000	87	2	2	2	2	1	2	2	0	1	0	0	0	0	$d_{z2} \rightarrow \pi_x$*
S_5	3.19	388	0.0000	39	2	2	2	1	2	2	2	0	0	1	0	0	0	$d_{yz} \rightarrow \pi_y$*
				39	2	2	1	2	2	2	2	0	1	0	0	0	0	$d_{xz} \rightarrow \pi_x$*
S_6	3.20	388	0.0000	43	2	2	1	2	2	2	2	0	1	0	0	0	0	$d_{xz} \rightarrow \pi_x$*
				42	2	2	2	1	2	2	2	0	0	1	0	0	0	$d_{yz} \rightarrow \pi_y$*
S_7	3.24	382	0.0000	39	2	2	2	1	2	2	2	0	1	0	0	0	0	$d_{yz} \rightarrow \pi_x$*
				39	2	2	1	2	2	2	2	0	0	1	0	0	0	$d_{xz} \rightarrow \pi_y$*
S_8	3.25	381	1.6489	42	2	2	2	2	2	1	2	0	1	0	0	0	0	$\pi_2 \rightarrow \pi_x$*
				26	2	2	2	2	2	2	1	0	0	1	0	0	0	$\pi_1 \rightarrow \pi_y$*
				12	2	2	2	2	2	1	2	0	0	1	0	0	0	$\pi_2 \rightarrow \pi_y$*
S_9	3.25	381	1.6491	42	2	2	2	2	2	1	2	0	0	1	0	0	0	$\pi_2 \rightarrow \pi_y$*
				26	2	2	2	2	2	2	1	0	1	0	0	0	0	$\pi_1 \rightarrow \pi_x$*
				12	2	2	2	2	2	1	2	0	1	0	0	0	0	$\pi_2 \rightarrow \pi_x$*
S_{10}	3.48	356	0.0000	41	2	2	1	2	2	2	2	0	0	1	0	0	0	$d_{xz} \rightarrow \pi_y$*
				41	2	2	2	1	2	2	2	0	1	0	0	0	0	$d_{yz} \rightarrow \pi_x$*
T_1	2.14	579		69	2	2	2	2	2	1	2	0	1	0	0	0	0	$\pi_2 \rightarrow \pi_x$*
				20	2	2	2	2	2	1	2	0	0	1	0	0	0	$\pi_2 \rightarrow \pi_y$*
T_2	2.14	579		69	2	2	2	2	2	1	2	0	0	1	0	0	0	$\pi_2 \rightarrow \pi_y$*
				20	2	2	2	2	2	1	2	0	1	0	0	0	0	$\pi_2 \rightarrow \pi_x$*
T_3	2.34	529		88	2	2	2	2	2	2	1	0	1	0	0	0	0	$\pi_1 \rightarrow \pi_x$*
T_4	2.34	529		88	2	2	2	2	2	2	1	0	0	1	0	0	0	$\pi_1 \rightarrow \pi_y$*
T_5	3.07	404		87	2	2	2	2	1	2	2	0	0	1	0	0	0	$d_{z2} \rightarrow \pi_y$*
T_6	3.07	404		87	2	2	2	2	1	2	2	0	1	0	0	0	0	$d_{z2} \rightarrow \pi_x$*
T_7	3.14	395		43	2	2	2	1	2	2	2	0	1	0	0	0	0	$d_{yz} \rightarrow \pi_x$*
				42	2	2	1	2	2	2	2	0	0	1	0	0	0	$d_{xz} \rightarrow \pi_y$*
T_8	3.15	393		40	2	2	1	2	2	2	2	0	1	0	0	0	0	$d_{xz} \rightarrow \pi_x$*
				40	2	2	2	1	2	2	2	0	0	1	0	0	0	$d_{yz} \rightarrow \pi_y$*
T_9	3.16	393		91	2	2	2	2	1	2	2	1	0	0	0	0	0	$d_{z2} \rightarrow d_{xy}$-n
T_{10}	3.17	391		40	2	2	1	2	2	2	2	0	0	1	0	0	0	$d_{xz} \rightarrow \pi_y$*
				40	2	2	2	1	2	2	2	0	1	0	0	0	0	$d_{yz} \rightarrow \pi_x$*
T_{11}	3.17	391		42	2	2	2	1	2	2	2	0	0	1	0	0	0	$d_{yz} \rightarrow \pi_y$*

Table S9. Dependence of the excitation energies on the model and solvent type for the S_Q and S_B states and the three lowest triplet states for nickel porphyrin and palladium porphyrin complexes (PBE0 functional. def2-tzvp basis set)

CPCM

NiPor/PBE0	S_Q(ev)	(nm)	S_B(eV)	(nm)	T_1(eV)	(nm)	T2(eV)	(nm)	T3(eV)	(nm)
Vaccum	2.61	474	4.03	308	0.28	4356	0.57	2159	0.57	2159
Cyclohexane 2.02	2.61	476	3.75	331	0.31	4003	0.59	2109	0.59	2109
Benzene 2.27	2.61	476	3.71	334	0.31	3951	0.59	2101	0.59	2101
Chloroform 4.81	2.61	475	3.75	331	0.33	3705	0.60	2063	0.60	2063
Acetone 20.7	2.61	474	3.80	327	0.35	3503	0.61	2029	0.61	2029
Benzonitrile 26.0	2.61	475	3.71	334	0.36	3490	0.61	2027	0.61	2027
Methanol 32.7	2.61	474	3.81	325	0.36	3481	0.61	2026	0.61	2026
Acetonitrile 37.5	2.61	474	3.81	326	0.36	3477	0.61	2025	0.61	2025
Water 80.1	2.61	474	3.81	325	0.36	3458	0.61	2022	0.61	2022

Pdpor/PBE0	S_Q(eV)	(nm)	S_B(eV)	(nm)	T_1, T_2(eV)	(nm)	T_3,T(eV)	(nm)
Vaccum	2.65	469	4.00	310	2.08	595	2.23	557
Cyclohexane 2.02	2.64	470	3.74	332	2.09	593	2.23	555
Benzene 2.27	2.64	470	3.70	335	2.09	593	2.23	555
Chloroform 4.81	2.64	469	3.73	332	2.09	592	2.24	554
Acetone 20.7	2.65	469	3.78	328	2.10	592	2.24	554
Benzonitrile 26.0	2.64	469	3.70	335	2.10	591	2.24	553
Methanol 32.7	2.65	469	3.80	326	2.10	591	2.24	553
Acetonitrile 37.5	2.65	469	3.79	327	2.10	591	2.24	553
Water 80.1	2.65	469	3.80	326	2.10	591	2.24	553

SMD

NiPor/PBE0	S_Q(eV)	nm	S_B(eV)		T_1(eV)	nm	T_2(eV)		T_3(eV)	nm
Cyclohexane 2.02	2.60	476	3.71	334	0.31	4046	0.59	2115	0.59	2115
Benzene 2.27	2.60	476	3.67	338	0.31	4000	0.59	2108	0.59	2108
Chloroform 4.81	2.61	476	3.71	334	0.33	3738	0.60	2066	0.60	2066
Acetone 20.7	2.61	475	3.77	329	0.35	3503	0.61	2025	0.61	2025
Benzonitrile 26.0	2.61	475	3.67	338	0.36	3490	0.61	2023	0.61	2023
Methanol 32.7	2.61	475	3.79	328	0.36	3461	0.61	2016	0.61	2016
Acetonitrile 37.5	2.61	475	3.78	328	0.36	3473	0.61	2020	0.61	2020
Water 80.1	2.61	475	3.78	328	0.36	3425	0.62	2009	0.62	2009

Pdpor/PBE0	SQ+(eV)	nm	S_B(eV)	nm	T_1. T_2(eV)	nm	T3. T4	nm
Cyclohexane 2.02	2.63	471	3.70	335	2.09	593	2.23	555
Benzene 2.27	2.63	471	3.66	339	2.09	593	2.24	555
Chloroform 4.81	2.64	470	3.70	335	2.09	592	2.24	554
Acetone 20.7	2.64	469	3.75	330	2.10	591	2.24	553
Benzonitrile 26.0	2.64	470	3.66	339	2.10	591	2.24	553
Methanol 32.7	2.64	469	3.77	329	2.10	591	2.24	553
Acetonitrile 37.5	2.64	469	3.76	330	2.10	591	2.24	553
Water 80.1	2.64	469	3.77	329	2.10	591	2.24	553

Table S10. Dependence of the excitation energy on the functional for the S_Q and S_B states and the three lowest triplets for nickel porphyrin and palladium porphyrin complexes. For hybrid functionals, the contribution of the HF exchange energy is shown.

NiPor	S_Q(eV)	(nm)	S_B(eV)	(nm)	T_1(eV)	(nm)	T_2(eV)	(nm)	T_3(nm)	(nm)
B3LYP (20%)	2.55	486	3.70	335	0.50	2461	0.75	1657	0.75	1657
PBE0 (25%)	2.61	474	3.81	325	0.36	3481	0.61	2026	0.61	2026
TPSSh (10%)	2.55	486	3.65	340	0.87	1421	1.09	1136	1.09	1136
TPSS0 (25%)	2.63	471	3.87	321	0.37	3316	0.67	1856	0.67	1856
M06 (27%)	2.52	493	3.72	333	0.40	3093	0.79	1566	0.79	1566
BP86	2.44	509	3.36	369	1.23	1012	1.32	942	1.32	942
PBE	2.44	507	3.35	370	1.22	1018	1.32	941	1.32	941
TPSS	2.48	500	3.39	365	1.23	1005	1.36	909	1.36	909
M06L	2.53	490	3.58	346	1.00	1243	1.40	883	1.40	883
CAM-B3LYP	2.61	475	3.92	316	0.43	2874	0.72	1734	0.72	1734
LC-PBE	2.67	464	4.17	297	0.72	1730	1.01	1226	1.01	1226
wB97X	2.57	482	3.98	312	0.39	3143	0.69	1808	0.69	1808
CAS/NEVPT2	2.39	517	3.20	387	0.23	5367	0.30	4202	0.30	4188
EXP	2.34	530	2.99	415						

Pdpor	S_Q(eV)	(nm)	S_B(eV)	(nm)	T_1,T_2(eV)	(nm)	T3,T4(eV)	(nm)
B3LYP	2.58	481	3.69	336	2.08	597	2.19	565
PBE0	2.65	469	3.80	326	2.10	591	2.24	553
TPSSh	2.58	481	3.66	339	2.08	597	2.20	563
TPSS0	2.66	466	3.85	322	2.10	591	2.26	549
M06	2.54	489	3.69	336	2.06	600	2.18	569
BP86	2.46	505	3.40	365	2.02	615	2.13	582
PBE	2.46	503	3.40	364	2.03	612	2.14	578
TPSS	2.50	496	3.49	356	2.04	607	2.17	572
M06L	2.54	488	3.59	346	2.10	590	2.21	561
CAM-B3LYP	2.64	469	3.91	317	2.04	607	2.30	538
LC-PBE	2.72	455	4.21	294	1.98	626	2.49	499
wB97X	2.61	475	3.98	311	1.97	629	2.35	528
CAS/NEVPT2	2.45	507	3.26	380	2.14	579	2.34	529
EXP	2.24	553	3.22	385				
	2.39	519						

CASSCF/NEVPT2 Calculations Details

Calculations were performed in def2-TZVP basis with the Effective Core Potential for the Pd atom. The CPCM solvent model was used. with methanol as the solvent. For NiPor. Calculations were performed using a CAS(13.14) active space (thirteen orbitals and fourteen electrons) and 50 singlet and 50 triplet states. For NiCorr. a CASSCF(12.12) active space with 35 singlet and 35 triplet states was used. For PdPor. a CASSCF(13.14) active space with 40 singlet and 40 triplet states was used. For PdCorr. a CASSCF(12.12) active space with 35 singlet and 35 triplet states was used. With fewer excited states included in the calculations. The lowest states exhibit large variation from case to case. This is because dynamical correlation has varying effects on the CASSCF states. and when it is included, the states are strongly rearranged. so that if there are too few of them, the low-lying electronic states do not appear. For example. for NiPor. the Soret band only appears after considering 50 excited states.

Supplementary Figures.

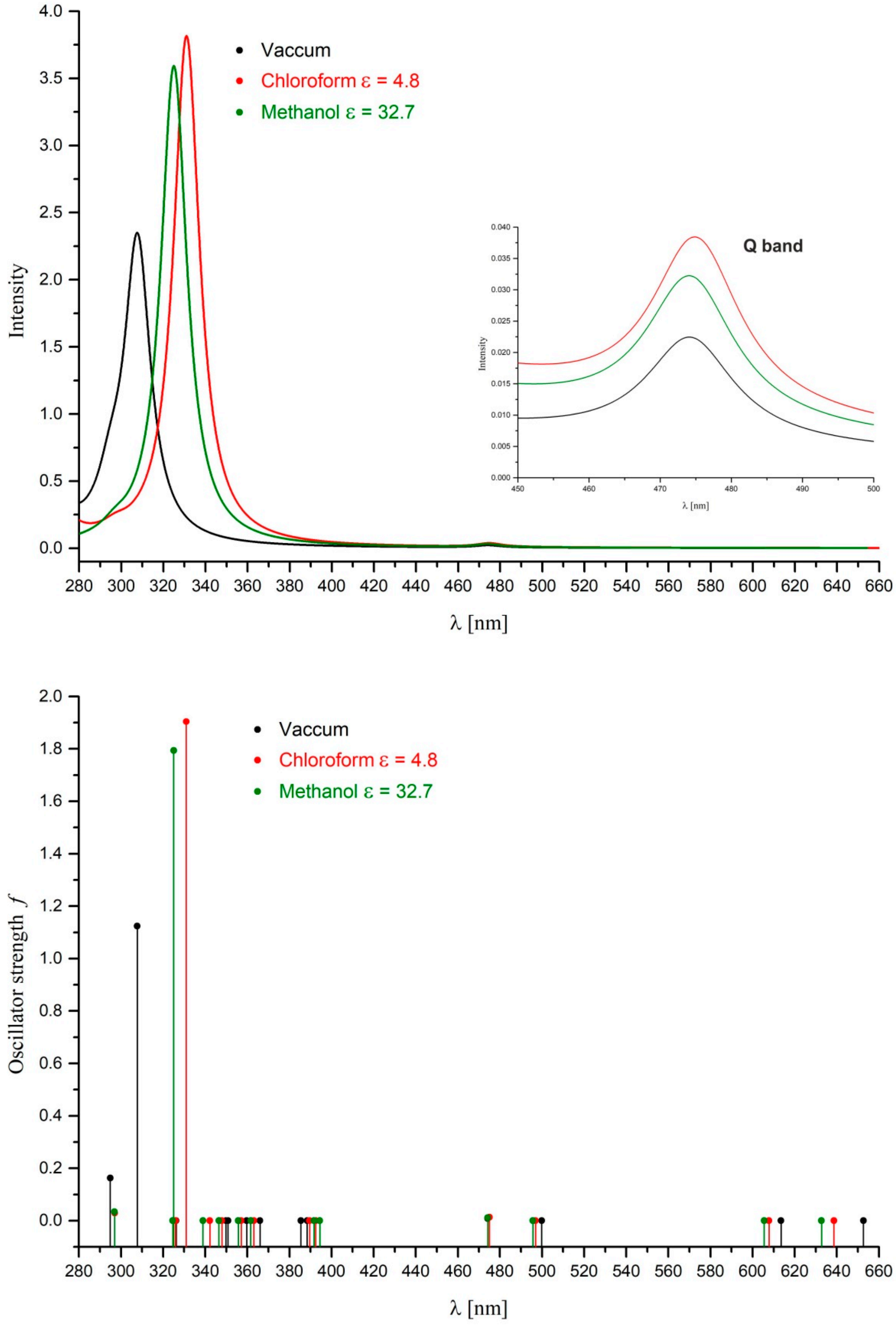

Figure S1. Upper panel: simulated UV/VIS spectra for the NiPor model complex obtained on the basis of the TDDFT/PBE0/def2-TZVP calculations with use CPCM solvation model and two different solvents. The simulated spectral lines were obtained using Lorentzian broadening with a half-width of 15 nm. Lower panel: calculated TDDFT wavelengths for vertical excitations to singlet states.

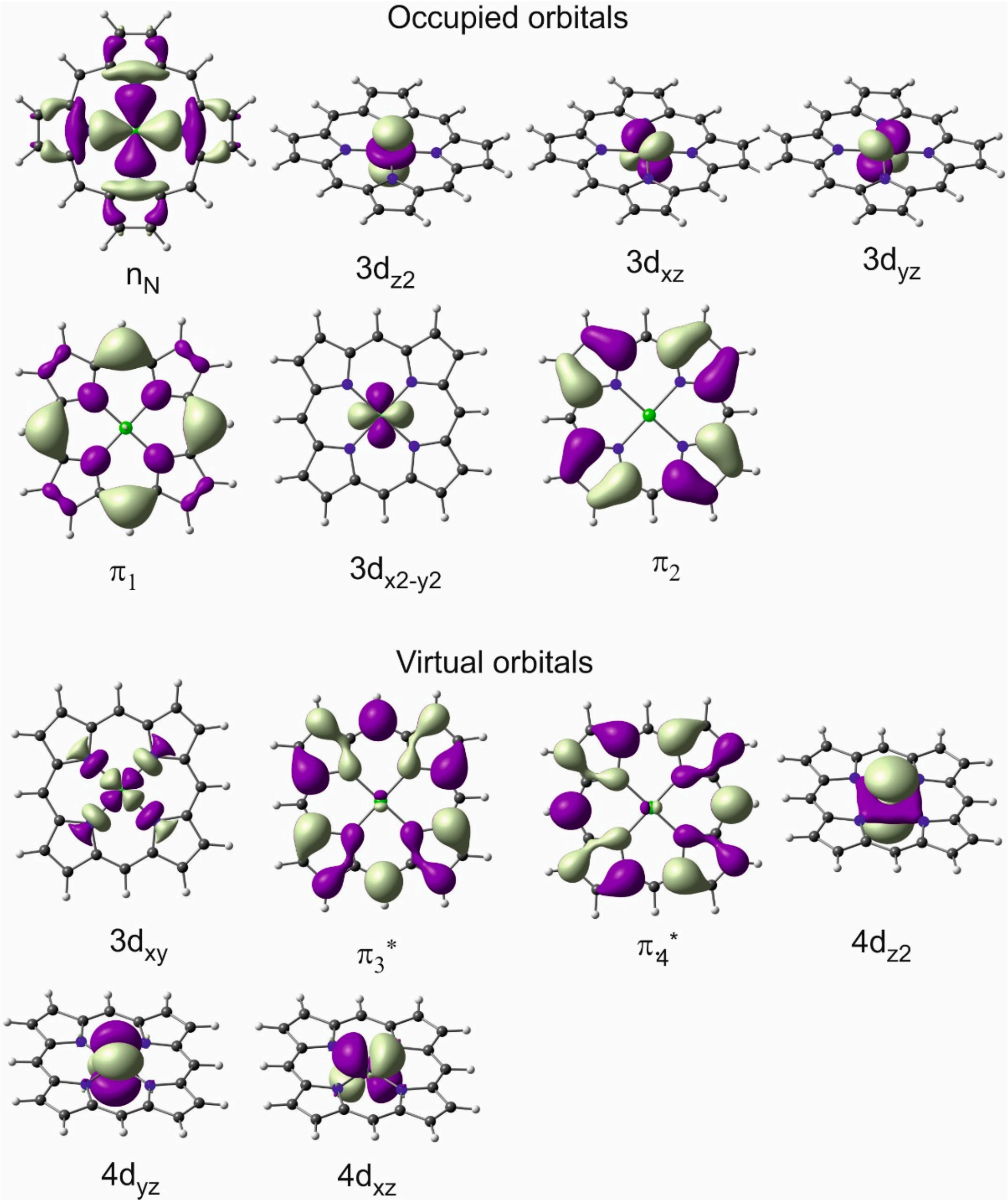

Figure S2. Natural orbitals composing active space CAS(14,13) in CASSCF/NEVPT2 calculations for NiPor complex.

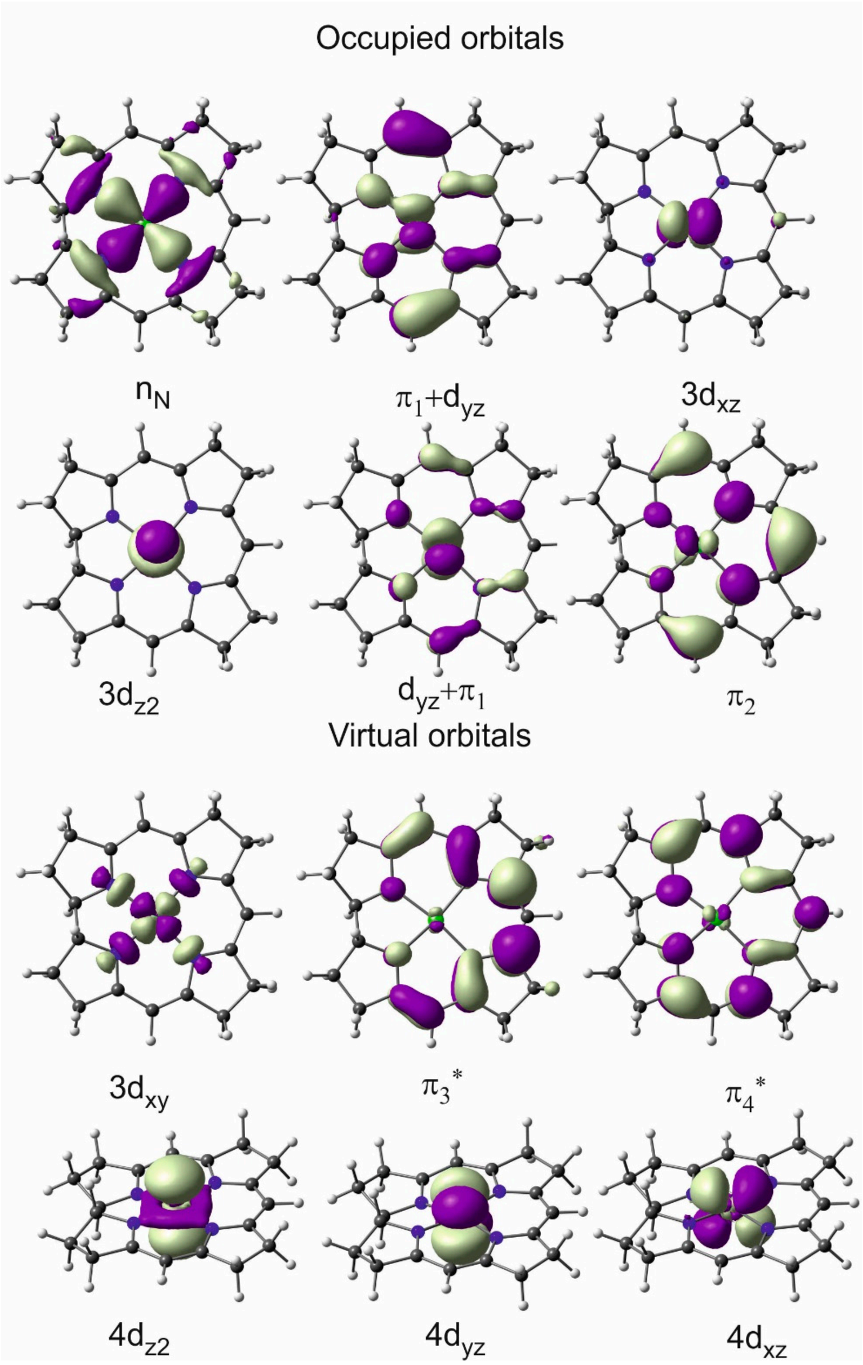

Figure S3. Natural orbitals composing active space CAS(12,12) in CASSCF/NEVPT2 calculations for NiCorr complex.

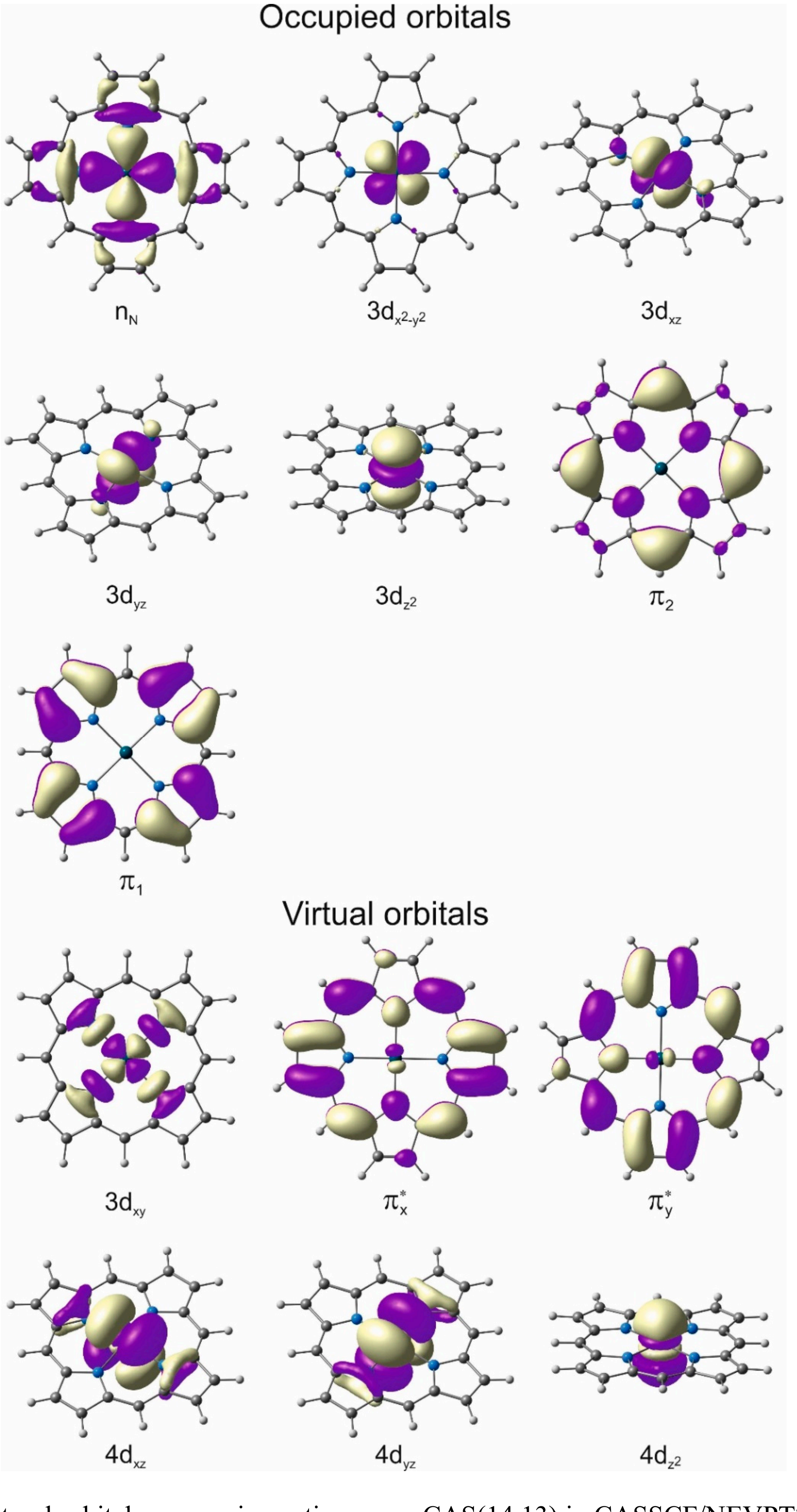

Figure S4. Natural orbitals composing active space CAS(14,13) in CASSCF/NEVPT2 calculations for PdPor complex.

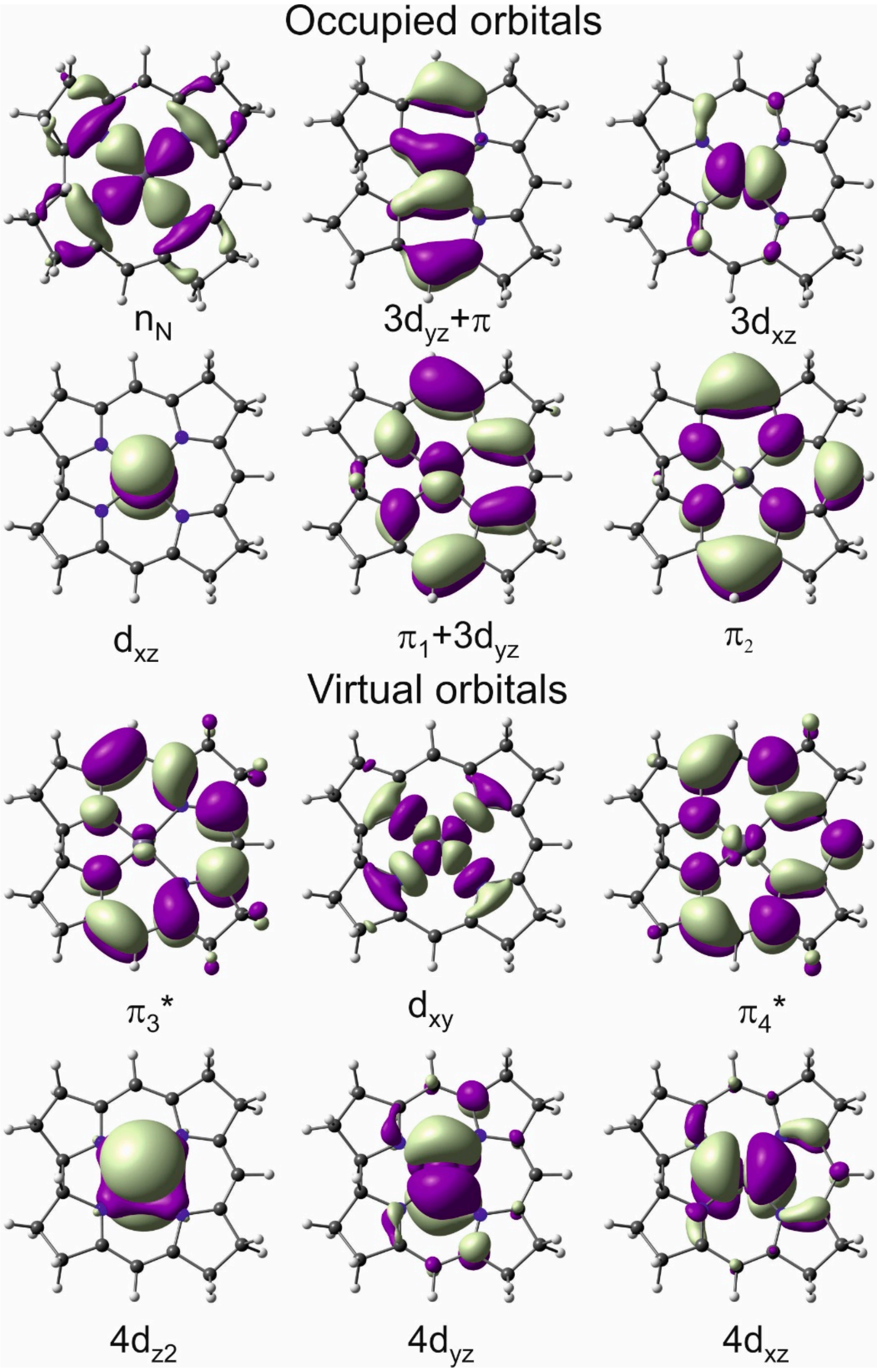

Figure S5. Natural orbitals composing active space CAS(12,12) in CASSCF/NEVPT2 calculations for PdCorr complex.

Optimized GS geometry NiPor, PBE0/def2-TZVP

C	8.957608000	-8.267529000	0.003862000
N	7.580988000	-6.252521000	0.000229000
N	7.592676000	-3.482122000	-0.000041000
N	10.363007000	-3.493924000	0.000084000
N	10.351407000	-6.264262000	-0.000146000
C	7.750613000	-7.611442000	0.002546000
C	6.488396000	-8.288259000	0.002171000
C	5.535952000	-7.327668000	-0.000962000
C	6.223545000	-6.071286000	-0.001884000
C	5.577690000	-4.858772000	-0.003746000
C	6.233756000	-3.651769000	-0.002396000
C	5.556933000	-2.389557000	-0.002025000
C	6.517513000	-1.437101000	0.001090000
C	7.773899000	-2.124681000	0.002008000
C	8.986429000	-1.478861000	0.003809000
C	10.193399000	-2.134985000	0.002396000
C	11.455619000	-1.458187000	0.001897000
C	12.408054000	-2.418783000	-0.001305000
C	11.720460000	-3.675161000	-0.002147000
C	12.366364000	-4.887647000	-0.004049000
C	11.710327000	-6.094665000	-0.002623000
C	12.387119000	-7.356891000	-0.002289000
C	11.426506000	-8.309314000	0.000932000
C	10.170135000	-7.621700000	0.001946000
H	8.952903000	-9.350310000	0.005538000
H	6.373458000	-9.362423000	0.003803000
H	4.460832000	-7.433387000	-0.002319000
H	4.494909000	-4.854090000	-0.005448000
H	4.482769000	-2.274624000	-0.003665000
H	6.411774000	-0.361982000	0.002418000
H	8.991171000	-0.396081000	0.005464000
H	11.570563000	-0.384024000	0.003503000
H	13.483175000	-2.313079000	-0.002757000
H	13.449144000	-4.892277000	-0.005847000
H	13.461278000	-7.471866000	-0.004024000
H	11.532211000	-9.384436000	0.002274000
Ni	8.972080000	-4.873109000	0.000154000

Optimized MECP geometry, NiPor PBE0/def2-TZVP

C	8.957498000	-8.285902000	-0.006944000
N	7.525047000	-6.307831000	-0.002448000
N	7.536971000	-3.425939000	0.004152000
N	10.418976000	-3.438577000	-0.003192000
N	10.407052000	-6.320502000	-0.006780000
C	7.719555000	-7.654624000	-0.005524000
C	6.449438000	-8.330170000	-0.005403000
C	5.493752000	-7.366336000	-0.001923000
C	6.179983000	-6.101930000	0.000095000
C	5.559285000	-4.858627000	0.005610000
C	6.190308000	-3.620596000	0.008694000
C	5.514851000	-2.350388000	0.016681000
C	6.478694000	-1.394716000	0.016646000
C	7.743100000	-2.080970000	0.008610000
C	8.986541000	-1.460534000	0.005307000
C	10.224461000	-2.091778000	-0.000375000
C	11.494539000	-1.416254000	-0.002294000
C	12.450246000	-2.380071000	-0.006151000
C	11.764044000	-3.644482000	-0.006375000
C	12.384749000	-4.887783000	-0.007634000
C	11.753728000	-6.125846000	-0.006486000
C	12.429228000	-7.396057000	-0.005337000
C	11.465394000	-8.351742000	-0.005072000
C	10.200958000	-7.665488000	-0.006136000
H	8.952795000	-9.370002000	-0.008246000
H	6.319879000	-9.403063000	-0.007671000
H	4.419828000	-7.486855000	-0.000814000
H	4.475212000	-4.853856000	0.008112000
H	4.441995000	-2.220450000	0.021543000
H	6.357832000	-0.320786000	0.021547000
H	8.991224000	-0.376435000	0.008061000
H	11.624101000	-0.343365000	-0.000717000
H	13.524166000	-2.259505000	-0.008470000
H	13.468827000	-4.892569000	-0.009171000
H	13.502104000	-7.525976000	-0.004923000
H	11.586265000	-9.425688000	-0.004290000
Ni	8.972037000	-4.873079000	-0.004236000

Optimized GS geometry NiCor PBE0/def2-TZVP

28	0.035336000	-0.000394000	-0.012513000
7	-1.353132000	1.206169000	-0.183514000
7	-1.352332000	-1.207804000	0.158971000
7	1.313227000	-1.396695000	-0.012465000
7	1.312381000	1.396752000	-0.013409000
6	-2.692510000	0.647533000	-0.396338000
1	-2.800079000	0.420607000	-1.465598000
6	1.025549000	-2.742151000	-0.068404000
6	-1.384207000	2.498432000	-0.128830000
6	2.643220000	-1.220922000	-0.018199000
6	-3.626973000	1.772941000	0.023409000
6	2.281769000	-3.548225000	-0.206862000
6	-2.784667000	3.022549000	-0.248586000
6	3.384225000	-2.519626000	-0.014026000
6	-0.217464000	-3.285978000	-0.026963000
6	-0.219467000	3.285086000	0.001390000
6	3.283282000	0.000614000	-0.013172000
6	-3.625906000	-1.775949000	-0.047034000
6	-2.782706000	-3.025096000	0.224344000
6	2.279713000	3.549018000	0.179354000
6	3.382812000	2.520876000	-0.012282000
6	-2.691956000	-0.649997000	0.372423000
1	-2.799123000	-0.423245000	1.441758000
6	-1.382623000	-2.500074000	0.104055000
6	1.023914000	2.742044000	0.042235000
6	2.642472000	1.221766000	-0.008008000
1	-3.838944000	-1.694277000	-1.116363000
1	-4.569841000	-1.766869000	0.495919000
1	-2.969290000	-3.850531000	-0.463548000
1	-2.929520000	-3.406483000	1.241093000
1	2.306717000	4.363054000	-0.544990000
1	2.311833000	3.998316000	1.175229000
1	3.897263000	2.634529000	-0.970727000
1	4.146475000	2.538191000	0.766138000
1	-0.317639000	-4.361333000	-0.086921000
1	-0.320262000	4.360397000	0.061068000
1	4.365350000	0.000956000	-0.013297000
1	-4.571169000	1.763222000	-0.519076000
1	-3.839396000	1.691301000	1.092864000
1	2.313971000	-3.995792000	-1.203529000
1	2.309465000	-4.363424000	0.516132000
1	-2.932163000	3.403622000	-1.265360000
1	-2.971494000	3.848053000	0.439157000
1	4.148662000	-2.536379000	-0.791687000
1	3.897951000	-2.633536000	0.944797000

Optimized MECP geometry for NiPor, PBE0/def2-TZVP

Ni	0.073077000	-0.026650000	-0.635740000
N	-1.441615000	1.256687000	-0.787236000
N	-1.321916000	-1.098924000	0.220027000
N	1.387396000	-1.471626000	-0.329150000
N	1.326821000	1.376728000	-0.082882000
C	-2.754932000	0.624793000	-0.699737000
H	-2.986864000	0.153871000	-1.660213000
C	1.059838000	-2.786462000	-0.132085000
C	-1.443694000	2.444177000	-0.280470000
C	2.700418000	-1.282133000	-0.189973000
C	-3.726523000	1.761668000	-0.358551000
C	2.305843000	-3.625911000	-0.158578000
C	-2.819112000	2.909898000	0.101888000
C	3.423764000	-2.589222000	-0.037901000
C	-0.206384000	-3.232757000	0.105180000
C	-0.259758000	3.208477000	-0.023160000
C	3.307250000	-0.034028000	-0.106147000
C	-3.615016000	-1.671100000	0.213372000
C	-2.745608000	-2.902413000	0.497649000
C	2.238340000	3.499761000	0.361162000
C	3.368588000	2.498428000	0.132945000
C	-2.641188000	-0.496328000	0.353433000
H	-2.704760000	-0.030432000	1.345773000
C	-1.353491000	-2.393348000	0.253104000
C	0.999625000	2.702644000	0.058757000
C	2.650421000	1.190534000	-0.027096000
H	-3.992818000	-1.711550000	-0.811467000
H	-4.468617000	-1.590050000	0.884623000
H	-2.981331000	-3.765318000	-0.126818000
H	-2.816577000	-3.229977000	1.540846000
H	2.306587000	4.384996000	-0.271057000
H	2.207774000	3.842037000	1.398814000
H	3.931898000	2.711271000	-0.780262000
H	4.089891000	2.451953000	0.949912000
H	-0.355020000	-4.297651000	0.231469000
H	-0.390025000	4.262700000	0.189209000
H	4.385926000	-0.013949000	-0.014712000
H	-4.289900000	2.049271000	-1.246252000
H	-4.443725000	1.466356000	0.408095000
H	2.357677000	-4.167316000	-1.107278000
H	2.311240000	-4.366457000	0.640706000
H	-3.058114000	3.868454000	-0.364182000
H	-2.847626000	3.070267000	1.183739000
H	4.202683000	-2.690194000	-0.795923000
H	3.918289000	-2.626187000	0.936108000

Optimized geometry og Pdpor GS, PBE0/def2-TZVP

C	8.957537000	-8.280303000	-0.000841000
N	7.541456000	-6.291518000	-0.001950000
N	7.553419000	-3.442546000	-0.000028000
N	10.402584000	-3.454893000	-0.002388000
N	10.390591000	-6.303895000	-0.001257000
C	7.733354000	-7.640857000	-0.002382000
C	6.465204000	-8.312326000	-0.003706000
C	5.511646000	-7.350638000	-0.003414000
C	6.193822000	-6.088231000	-0.001957000
C	5.564806000	-4.858657000	0.000316000
C	6.204064000	-3.634405000	0.002171000
C	5.532793000	-2.366341000	0.005855000
C	6.494524000	-1.412808000	0.005825000
C	7.756799000	-2.094896000	0.002121000
C	8.986486000	-1.466145000	0.000242000
C	10.210675000	-2.105563000	-0.002156000
C	11.478843000	-1.434088000	-0.003625000
C	12.432401000	-2.395759000	-0.004139000
C	11.750223000	-3.658194000	-0.003000000
C	12.379217000	-4.887766000	-0.001501000
C	11.739947000	-6.112022000	0.000258000
C	12.411230000	-7.380078000	0.003157000
C	11.449511000	-8.333626000	0.003348000
C	10.187235000	-7.651545000	0.000549000
H	8.952668000	-9.363440000	-0.000529000
H	6.343225000	-9.385688000	-0.004515000
H	4.437305000	-7.463604000	-0.003921000
H	4.481668000	-4.854100000	0.000929000
H	4.459508000	-2.244013000	0.008062000
H	6.381327000	-0.338519000	0.007993000
H	8.991355000	-0.383007000	0.000951000
H	11.600854000	-0.360728000	-0.004004000
H	13.506747000	-2.282778000	-0.005000000
H	13.462358000	-4.892312000	-0.001373000
H	13.484518000	-7.502395000	0.004736000
H	11.562714000	-9.407916000	0.005092000
Pd	8.972045000	-4.873181000	-0.001465000

Optimized MECP geometry of PdPor, PBE0/def2-TZVP

C	9.026071000	-8.280009000	-0.034923000
N	7.634051000	-6.199701000	-0.015961000
N	7.418345000	-3.306210000	-0.301151000
N	10.309989000	-3.546509000	-0.015905000
N	10.524194000	-6.438739000	-0.312779000
C	7.835013000	-7.558214000	0.047934000
C	6.568362000	-8.212054000	0.199725000
C	5.613456000	-7.249042000	0.202734000
C	6.277481000	-5.987451000	0.053024000
C	5.565491000	-4.790042000	-0.022899000
C	6.096754000	-3.514990000	-0.158351000
C	5.412320000	-2.247821000	-0.011262000
C	6.376814000	-1.291490000	-0.010560000
C	7.638224000	-1.986547000	-0.157293000
C	8.917756000	-1.466179000	-0.021152000
C	10.109194000	-2.188259000	0.054090000
C	11.376446000	-1.535024000	0.203451000
C	12.331344000	-2.498059000	0.199003000
C	11.666763000	-3.759071000	0.046920000
C	12.378388000	-4.956214000	-0.036626000
C	11.846432000	-6.230708000	-0.175024000
C	12.531488000	-7.498538000	-0.036595000
C	11.566969000	-8.454822000	-0.035980000
C	10.304952000	-7.759026000	-0.174028000
H	8.926533000	-9.354898000	0.065968000
H	6.444437000	-9.282224000	0.284033000
H	4.542545000	-7.364200000	0.289988000
H	4.490150000	-4.881028000	0.081159000
H	4.346765000	-2.119362000	0.117005000
H	6.257342000	-0.224980000	0.118468000
H	9.017698000	-0.391725000	0.083856000
H	11.500713000	-0.465204000	0.291459000
H	13.402579000	-2.383259000	0.282568000
H	13.454147000	-4.865665000	0.063328000
H	13.597591000	-7.627616000	0.086362000
H	11.686980000	-9.521912000	0.087665000
Pd	8.969482000	-4.870506000	-1.020102000

Optimized GS geometry of PdCorr, PBE0/def2-TZVP

Pd	0.039172000	-0.001103000	-0.013436000
N	-1.424299000	1.249125000	-0.151460000
N	-1.423565000	-1.252105000	0.124305000
N	1.377389000	-1.445675000	0.022872000
N	1.376772000	1.444379000	-0.049492000
C	-2.759738000	0.679452000	-0.357728000
H	-2.887095000	0.518029000	-1.436694000
C	1.053463000	-2.776839000	-0.019397000
C	-1.414861000	2.537406000	-0.083659000
C	2.698209000	-1.243679000	0.003716000
C	-3.663749000	1.805665000	0.133696000
C	2.315538000	-3.590862000	-0.085607000
C	-2.822148000	3.065316000	-0.139748000
C	3.432358000	-2.550383000	0.006390000
C	-0.210598000	-3.296905000	-0.023801000
C	-0.212374000	3.294526000	-0.002527000
C	3.316935000	-0.000085000	-0.014319000
C	-3.662869000	-1.809686000	-0.160136000
C	-2.820608000	-3.068942000	0.113176000
C	2.313559000	3.589824000	0.062647000
C	3.430779000	2.550593000	-0.037448000
C	-2.759251000	-0.683031000	0.330965000
H	-2.886228000	-0.521726000	1.409988000
C	-1.413600000	-2.540366000	0.056871000
C	1.051921000	2.775324000	-0.006449000
C	2.697739000	1.243303000	-0.031848000
H	-3.829734000	-1.700962000	-1.235159000
H	-4.629286000	-1.827424000	0.341412000
H	-2.985276000	-3.873691000	-0.604159000
H	-3.009869000	-3.476154000	1.112439000
H	2.342677000	4.326741000	-0.740957000
H	2.347776000	4.139159000	1.005950000
H	4.006755000	2.644229000	-0.961582000
H	4.141705000	2.598911000	0.788979000
H	-0.303448000	-4.374023000	-0.080191000
H	-0.305940000	4.371559000	0.054504000
H	4.399864000	0.000071000	-0.015089000
H	-4.630308000	1.823039000	-0.367587000
H	-3.830267000	1.696768000	1.208758000
H	2.348851000	-4.147537000	-1.024537000
H	2.345939000	-4.321588000	0.723683000
H	-3.011825000	3.472370000	-1.138996000
H	-2.987154000	3.869971000	0.577610000
H	4.136908000	-2.598458000	-0.825595000
H	4.015717000	-2.642846000	0.925891000

Optimized MECP geometry of PdCOrr, PBE0/def2-TZPV

Pd	0.112208000	-0.023072000	-1.356393000
N	-1.365784000	1.158453000	-0.429974000
N	-1.517910000	-1.408300000	-0.439944000
N	1.216526000	-1.402206000	-0.210928000
N	1.445750000	1.550230000	-0.417104000
C	-2.745133000	0.625611000	-0.458450000
H	-3.051676000	0.528651000	-1.508457000
C	0.928767000	-2.765823000	-0.203053000
C	-1.335208000	2.435115000	-0.196468000
C	2.527988000	-1.182732000	0.006735000
C	-3.549024000	1.704384000	0.254396000
C	2.223766000	-3.529319000	-0.164447000
C	-2.730298000	2.970856000	-0.004223000
C	3.238792000	-2.481282000	0.263166000
C	-0.287298000	-3.379042000	-0.207100000
C	-0.183663000	3.273633000	-0.109002000
C	3.218691000	0.022354000	0.068239000
C	-3.721822000	-1.833653000	0.147644000
C	-2.853915000	-3.079881000	0.429026000
C	2.348183000	3.653406000	0.105365000
C	3.415828000	2.575994000	0.319460000
C	-2.652203000	-0.747533000	0.173045000
H	-2.381954000	-0.566238000	1.226952000
C	-1.518828000	-2.650211000	-0.137682000
C	1.109979000	2.844206000	-0.175691000
C	2.692089000	1.308286000	-0.045948000
H	-4.170754000	-1.901186000	-0.847585000
H	-4.512799000	-1.689037000	0.883363000
H	-3.224207000	-3.998675000	-0.029409000
H	-2.757895000	-3.267370000	1.504980000
H	2.575217000	4.288239000	-0.755427000
H	2.216179000	4.307917000	0.966886000
H	4.302031000	2.705560000	-0.305267000
H	3.758610000	2.529735000	1.355983000
H	-0.296428000	-4.460431000	-0.146025000
H	-0.367007000	4.316407000	0.116884000
H	4.269108000	-0.057785000	0.320658000
H	-4.574146000	1.772778000	-0.107439000
H	-3.574016000	1.490117000	1.326673000
H	2.445150000	-3.896608000	-1.171296000
H	2.167571000	-4.391744000	0.498280000
H	-3.036418000	3.474577000	-0.928302000
H	-2.776149000	3.707106000	0.799048000
H	4.187057000	-2.528948000	-0.271864000
H	3.462344000	-2.546003000	1.332831000
